# Chromosome 8 gain is associated with high-grade transformation in MPNST

**DOI:** 10.1172/jci.insight.146351

**Published:** 2021-03-22

**Authors:** Carina Dehner, Chang In Moon, Xiyuan Zhang, Zhaohe Zhou, Chris Miller, Hua Xu, Xiaodan Wan, Kuangying Yang, Jay Mashl, Sara J.C. Gosline, Yuxi Wang, Xiaochun Zhang, Abigail Godec, Paul A. Jones, Sonika Dahiya, Himanshi Bhatia, Tina Primeau, Shunqiang Li, Kai Pollard, Fausto J. Rodriguez, Li Ding, Christine A. Pratilas, Jack F. Shern, Angela C. Hirbe

**Affiliations:** 1 Department of Pathology and Immunology and; 2 Department of Internal Medicine, Division of Oncology, Washington University in St. Louis, St. Louis, Missouri, USA.; 3 Pediatric Oncology Branch, Center for Cancer Research, National Cancer Institute, NIH, Bethesda, Maryland, USA.; 4 McDonnell Genome Institute, Washington University in St. Louis, St. Louis, Missouri, USA.; 5 The First Affiliated Hospital, Nanchang University, Nangchang, China.; 6 Pacific Northwest National Laboratory, Seattle, Washington, USA.; 7 Siteman Cancer Center Division of Pediatric Oncology, St. Louis, Missouri, USA.; 8 Division of Pediatric Oncology, The Sidney Kimmel Comprehensive Cancer Center at Johns Hopkins, Baltimore, Maryland, USA.; 9 Department of Pathology, John Hopkins University, Baltimore, Maryland, USA

**Keywords:** Genetics, Oncology, Cancer

## Abstract

One of the most common malignancies affecting adults with Neurofibromatosis type 1 (NF1) is the malignant peripheral nerve sheath tumor (MPNST), an aggressive and often fatal sarcoma that commonly arises from benign plexiform neurofibromas. Despite advances in our understanding of MPNST pathobiology, there are few effective therapeutic options, and no investigational agents have proven successful in clinical trials. To further understand the genomic heterogeneity of MPNST, and to generate a preclinical platform that encompasses this heterogeneity, we developed a collection of NF1-MPNST patient-derived xenografts (PDX). These PDX were compared with the primary tumors from which they were derived using copy number analysis, whole exome sequencing, and RNA sequencing. We identified chromosome 8 gain as a recurrent genomic event in MPNST and validated its occurrence by FISH in the PDX and parental tumors, in a validation cohort, and by single-cell sequencing in the PDX. Finally, we show that chromosome 8 gain is associated with inferior overall survival in soft-tissue sarcomas. These data suggest that chromosome 8 gain is a critical event in MPNST pathogenesis and may account for the aggressive nature and poor outcomes in this sarcoma subtype.

## Graphical abstract

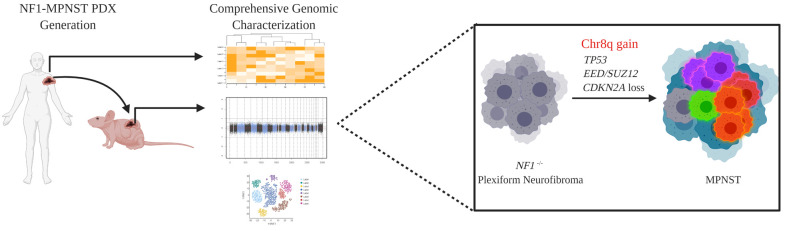

## Introduction

Malignant peripheral nerve sheath tumors (MPNST) are aggressive soft-tissue sarcomas with a devastating prognosis. About half of MPNST arise in patients with an underlying autosomal dominant genetic disorder, Neurofibromatosis type 1 (NF1), while the other half arise de novo or in the setting of prior radiation therapy (1–3). NF1 is one of the most common genetic syndromes, with an annual incidence of 1 in 2500 individuals world-wide; the risk of MPNST in a genetically predisposed individual is about 8%–13% (4). In the context of NF1, MPNST are most often the result of malignant transformation of benign precursor lesions, plexiform neurofibroma (PN) or atypical neurofibromatous neoplasms of uncertain biological potential (ANNUBP), previously called atypical neurofibroma (AN) (5).

Critical inactivating mutations in the *NF1* gene, and consequent hyperactive RAS signaling, are seen in sporadic as well as NF1-associated MPNST (6, 7). Mutations in other genes such as *TP53*, *EED*, *SUZ12*, and *CDKN2A* are required for malignant transformation to MPNST (8, 9). Despite advances in understanding the oncogenic drivers of MPNST, chemotherapy is inconsistently beneficial, and other effective therapies are lacking. The only curative option is aggressive local control with surgery and radiation therapy. Advanced disease often follows a rapidly progressing course, and metastases to bone and lungs are common complications (1). To date, no clinical trial has been effective for treatment in the advanced or metastatic setting (10–12). Five-year overall survival (OS) in patients with NF1-MPNST is dismal, at 20%–50% (13). Numerous in vivo tumor models have been developed for preclinical studies, but there is limited genetic diversity modeled in these systems, and they do not capture the full spectrum of genetic heterogeneity found in human NF1-MPNST. *TP53* loss, for example, is a common feature of the currently used preclinical models, but it is observed only in a minority of human MPNST (14, 15).

To address this critical problem, we have successfully generated a series of NF1-MPNST patient-derived xenografts (PDX) that more accurately reflect the genetic heterogeneity seen in the human condition. Patient tumor tissue is implanted directly into immunodeficient mice; in vivo tumors are grown and serially passaged (16). These models better reflect the genomic and phenotypic heterogeneity of cancer (14). Here, we present the development and comprehensive genomic characterization of 8 NF1-MPNST PDX suitable as preclinical tools in order to further study MPNST pathogenesis by identifying other drivers of transformation. In addition, we present the first use, to our knowledge, of single-cell RNA sequencing (scRNA-seq) of NF1-MPNST (16, 17). Through characterization of these models, we identified a gain in the long arm of chromosome 8 (Chr8q) as a nearly universal event in MPNST. Further, Chr8q gain is associated with inferior outcomes in soft-tissue sarcomas, which may contribute to the dismal OS of MPNST.

## Results

### Global characterization of PDX lines.

**Figure 1:**
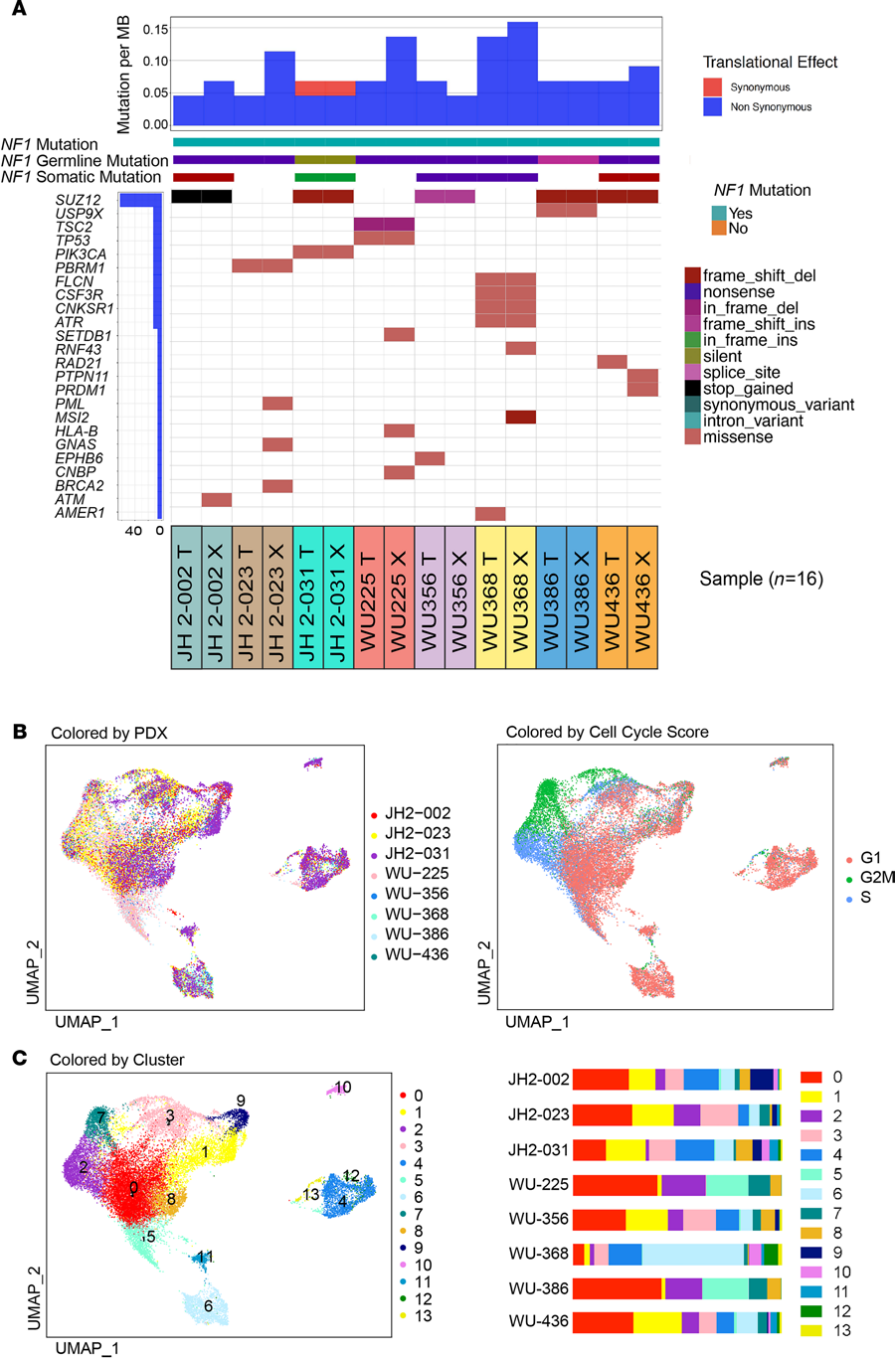
Whole exome sequencing and single-cell composition of 8 tumor-PDX pairs highlights intertumor heterogeneity. (**A**) Somatic variants across samples. Distinct somatic variant signatures are appreciated in each tumor-PDX pair. (**B**) Uniform Manifold Approximation and Projection (UMAP) was used as a dimension reduction tool to visualize the complex connections among the cells in the scRNA-seq result. Distribution of the 8 MPNST PDX scRNA-seq results is shown in the UMAP as colored by each PDX (left panel). Annotation of each cell’s predicted cell cycle state is shown in the UMAP (right panel). (**C**) UMAP of all PDX shows each cluster, and the bar plot demonstrates the intertumor heterogeneity across PDX, since each line is composed of varying percentages of the 14 different clusters.

Eight MPNST PDX lines were established from 8 biopsy-proven NF1-MPNST between 2014 and 2019 at 2 different institutions: Washington University and John Hopkins University. The engraftment success rate was 50%. Whole exome sequencing (WES) and bulk RNA-seq were carried out at the McDonnel Genome Institute at Washington University. Clinical parameters are summarized in Table 1. All tumors were high grade and greater than 5 centimeters. Seven of the patients were male, with a median age of onset of 30.5 years (range, 9–49 years). All PDX are histologically comparable with the parental tumors based on morphology on H&E stains and S100, COL4A, and KI-67 staining (Supplemental Figure 1; supplemental material available online with this article; https://doi.org/10.1172/jci.insight.146351DS1). Our somatic variant analysis identified an average of 80 high-confidence single-nucleotide variants (SNV) and short indels per sample (range, 13–99). Germline and common variants based on the 1000 genomes project (minor allele frequency [MAF] > 0.05) were filtered out. As expected based on prior genomic studies, *TP53* mutations occurred in 12.5% (1 of 8 pairs) and *SUZ12* mutations in 62.5% (5 of 8 pairs) of patients. Immunostaining revealed that H3K27me3 was absent, consistent with *SUZ12* mutations that we observed in these samples (18) (Supplemental Figure 1B). The other 25% (2 of 8 pairs) had mutations in other cancer-related pathways, such as DNA repair genes. The average mutation burden is 0.08 (range, 0.045–0.16) mutations/megabase (Figure 1A). Correlation of the variant allele frequency (VAF) in the parental tumor versus the VAF in each PDX demonstrated similarity of the PDX compared with its parental tumor. Interestingly, some variants showed a VAF difference, likely representing variants found predominantly in either the parental tumor or the PDX, suggesting that some clonal selection occurs upon engraftment (Supplemental Figure 2). Engraftment-related selection is also illustrated by the fact that some genes were found to have somatic variants in the PDX but not in the parental tumor (Figure 1A), with a median correlation coefficient of 0.474 (range, 0.014–0.791). This finding is also in line with other publications, which have shown an increased tumor burden in PDX compared with parental tumors (19). Nonetheless, scatter plots comparing the gene expression levels in the parental tumor versus the PDX showed an overall similarity, with a median correlation coefficient of 0.79 (range, 0.66–0.92) (Supplemental Figure 3). Hierarchical clustering by Euclidean distance and *Z* scores was performed on all expressed genes in parental tumors and PDX samples, further highlighting the intertumoral heterogeneity (Supplemental Figure 4).

To characterize the intratumoral heterogeneity at single-cell resolution, we performed droplet-based scRNA-seq on 24,055 cells from each of the 8 MPNST PDX (Supplemental Table 1). Focusing on the expansion of human MPNST in our mouse model, downstream analysis used only sequencing reads that uniquely aligned to the human genome (GRCh38). Clustering of the scRNA-seq data revealed that cells from different PDX shared transcriptional similarities, regardless of their cell cycle stages (Figure 1B), separating the cells into 14 distinct clusters, with each PDX contributing a unique distribution of cells to each cluster (Figure 1C and Supplemental Table 2).

### CNV analysis by whole genome sequencing (WGS) and scRNA-seq demonstrated Chr8q gain in MPNST.

**Figure 2:**
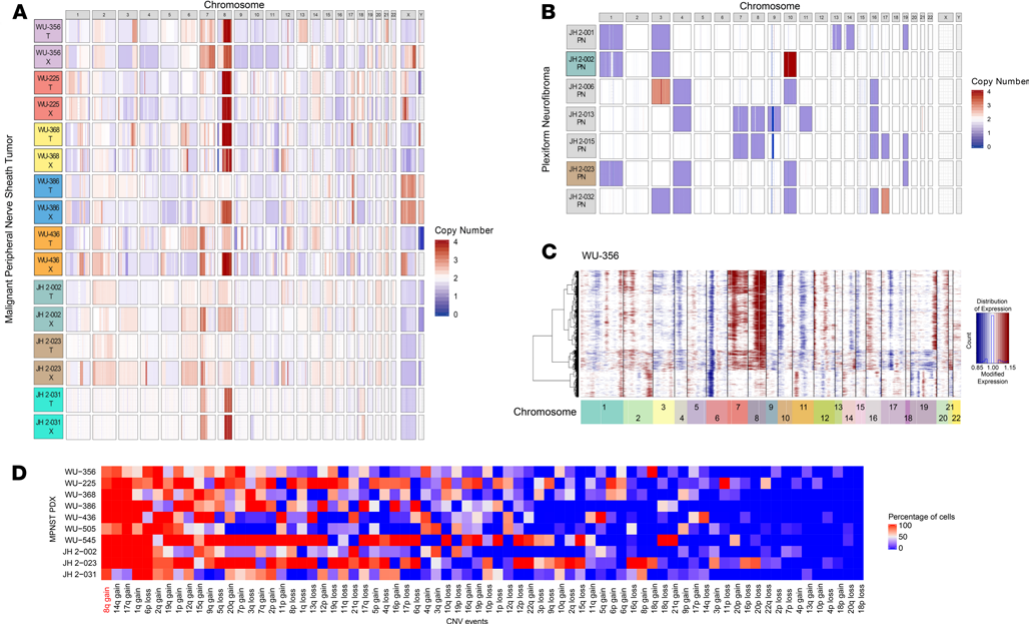
Chr8q gain is the most prevalent copy number variation in MPNST PDX. (**A**) Copy number variation (CNV) plot of all 8 MPNST PDX pairs. (**B**) CNV plot of all 7 PN samples. (**C**) Representative CNV heatmap with hierarchical clustering of results from inferCNV analysis of scRNA-seq result of WU-356. (**D**) Summary heatmap of all large -cale CNV events detected by inferCNV analysis of scRNA-seq data of all 8 MPNST PDX. Chr8q gain is the most prevalent CNV event among the 74 detected events.

In addition to coding sequence mutations and transcriptional expression, copy number variation (CNV) is another important source of genomic heterogeneity. We therefore analyzed the CNV profiles of the PDX, shown in Figure 2A, and found remarkable similarities with the corresponding patient tumors. Notably, 87% of the cases showed a Chr8q gain. To determine if Chr8q gain could occur early in tumor development, we performed CNV analysis on 7 PN, a precursor lesion of MPNST. Chr8q gain was not observed in any of the PN examined (Figure 2B). We also found a high degree of aneuploidy in NF1-MPNST, whereas the PN samples harbored diploid genomes, as expected based on prior publications (20–22).

The presence of CNV allowed us an opportunity to examine the clonal structure of each PDX generated with single-cell data using the inferCNV method (23, 24). As expected, all PDX exhibited a Chr8q gain in all or a high percentage of the tumor cells (Figure 2C and Supplemental Figure 5). A summary of each possible CNV event and the percentage of cells exhibiting these CNV events confirmed that Chr8q gain was the most prevalent and dominant CNV, present in > 90% of cells (Figure 2D, Table 2, and Supplemental Table 3). To our knowledge, these data illustrate the first use of single-cell sequencing in MPNST and demonstrate that Chr8q gain is an early, and highly recurrent, CNV event among the 74 events detected in all 8 NF1-MPNST.

**Figure 3:**
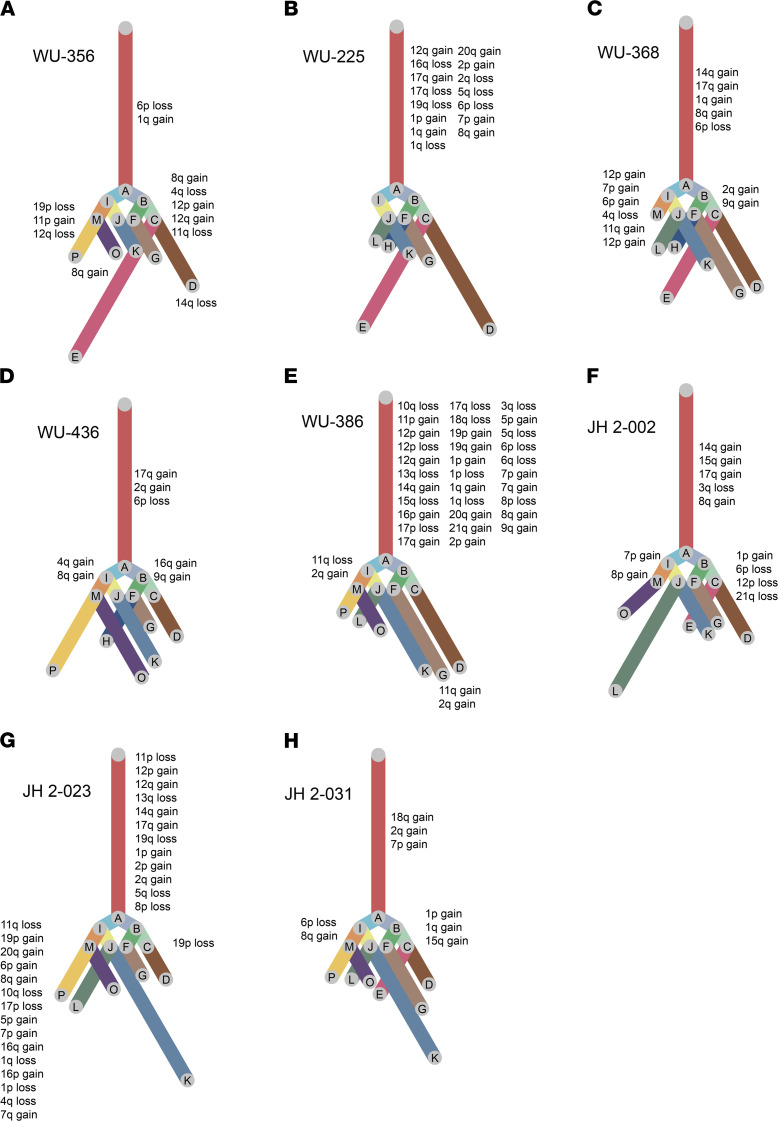
Clonal analysis of inferred CNV from scRNA-seq highlight complexity in clonal evolution and depict Chr8q gain as an early CNV in MPNST PDX. (**A–H**) Clonality tree of each of the 8 MPNST PDX. Lengths of the branches are scaled to the percentage of cells present in subclone of the corresponding CNV event. Copy number events appearing in 100% of the cells are depicted in these clonality plots.

Our analysis also revealed an underappreciated complexity of clonal evolution of MPNST PDX in the mouse model. For instance, in WU-356 — while Chr8q gain was a prominent event, along with 1q gain and 6p loss — we also appreciated the branching and evolution of subclone CNV (e.g., 11p gain, 12q loss, 19p loss) in 21% of all cells (Figure 2C and Figure 3A). Distinct patterns of genomic evolution shown by subclonal CNV events in each PDX indicate their evolutionary complexity in our model (Figure 3), as well as in the primary tumors. In addition to being the most common event (found in all MPNST PDX; Table 2), Chr8q gain appears to be an early copy number event, since it appears in the trunk of 5 of 8 PDX. While previous studies showed that every chromosome can be involved in numerical and/or structural aberrations in MPNST (21), one study did report a Chr8 gain accompanied by alterations in several other chromosomes by array comparative genomic hybridization (CGH) (25). Our results specifically support Chr8q gain and suggest that Chr8q gain may be important for high-grade transformation into MPNST.

### Chr8q gain is associated with MPNST transformation and poor OS in soft-tissue sarcomas.

**Figure 4:**
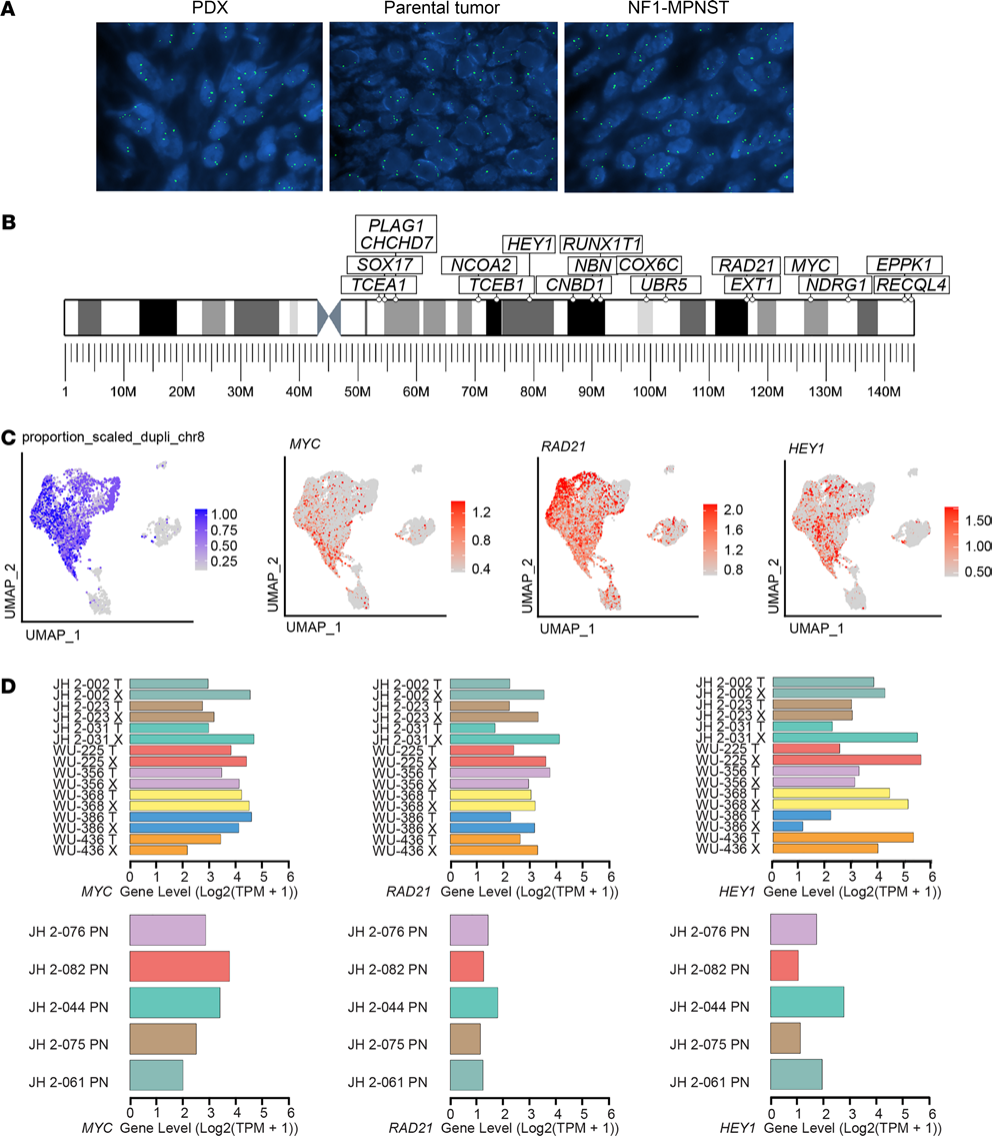
Chr8 gain is associated with MPNST transformation and inferior survival. (**A**) Representative photographs of fluorescence in situ hybridization (26) signals per nucleus of PDX, parental tumor, and control NF1-MPNST. (**B**) Important cancer-related genes on Chr8q. (**C**) Expression levels of the top 3 genes in each PDX-tumor pair and compared with PN. (**D**) UMAP of PDXs highlighting the existence of Chr8q gain and expression of *HEY1*, *MYC*, and *RAD21*.

To further validate the finding of Chr8q gain, we utilized fluorescence in situ hybridization (26), which permits the highly sensitive investigation of numerical chromosomal anomalies and genetic alterations on an individual-cell basis within a region of interest (ROI) (Figure 4A and Table 3) (27). Chr8 spans about 145 million bp, and 16% of its genes are implicated in cancer (28). Figure 4B depicts cancer-related genes in the area of Chr8q gain. Chr8 alterations are implicated in multiple malignancies, including translocations involving *MYC* in Burkitt lymphoma (29). Additionally, certain solid tumors show extra copies of Chr8, such as desmoid fibromatosis (30) and lipoblastoma (31, 32). There are increasing data showing that chromosomal gains can contribute to cancer pathogenesis, in addition to amplification of specific genes or chromosomal rearrangements (25, 33–35). Centromeric probe Chr8 fluorescence in situ hybridization (FISH) was performed on the 8 PDX models and 8 corresponding patient tumors, as well as 10 additional NF1-MPNST cases that served as a validation cohort, with 200 cells counted per case. All but 1 PDX showed Chr8 gain, defined as 3 or more copies, in > 50% of cells (7 of 8 PDX). Of the corresponding parental tumors, Chr8 gain was observed in 5 cases. However, all of the other cases (both PDX and parental tumors) demonstrated Chr8 gain in at least 10% of cells (Figure 4A and Table 3). These data suggest a selective advantage for cells with Chr8 gain during the engraftment process. Similarly, in the validation cohort, 8 of 10 NF1-MPNST had Chr8 gains in > 50% of cells, and in the remaining 2, Chr8 gains were present in at least 10% of cells. These findings strongly support the notion that Chr8 gain is a nearly universal event in MPNST.

**Figure 5:**
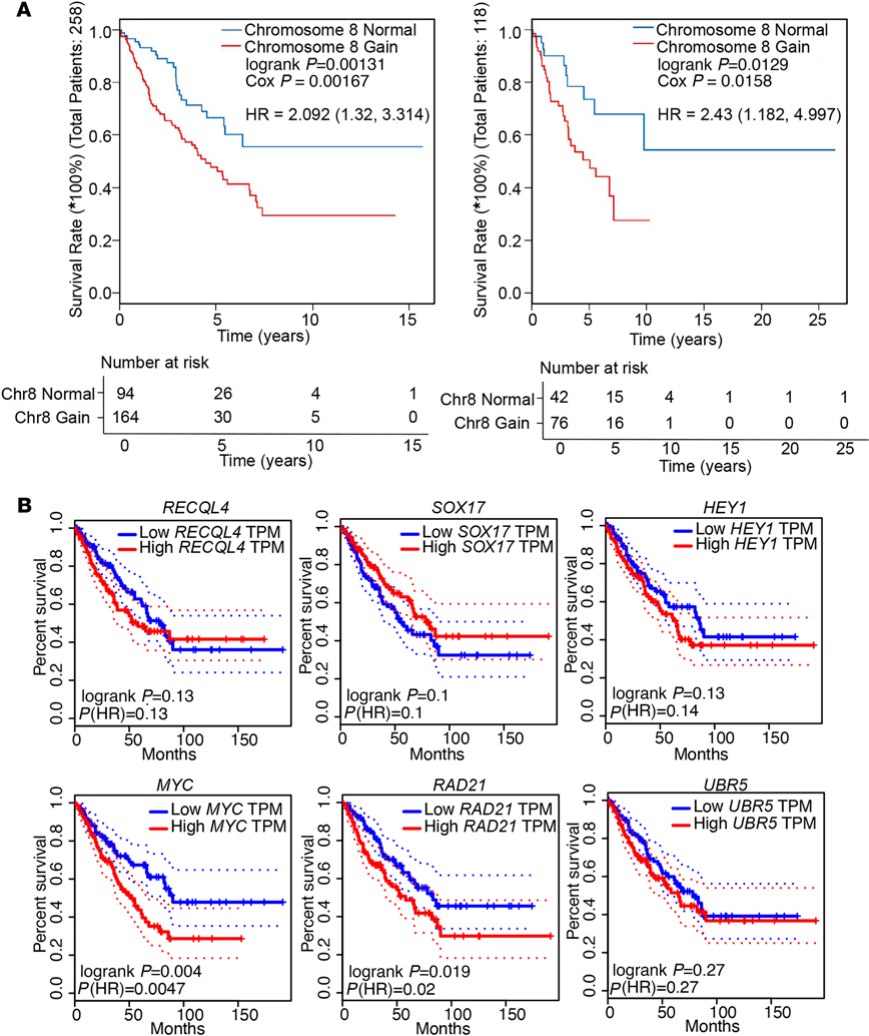
Survival analysis based on gene expression. (**A**) Kaplan-Meir curves showing the association of Chr8q gain with inferior overall survival in patients with soft-tissue sarcomas. Significance was assessed by log-rank test and the Cox proportional hazards model. (**B**) Survival analysis showing difference in overall survival in case of increased expression of *RECQL4*, *SOX17*, *HEY1*, *MYC*, *RAD21*, or *UBR5*. TPM, transcripts per million. Significance was assessed with the Cox proportional hazards model.

We next investigated the specific cancer-related genes located on Chr8q using an internal database of cancer-related genes, as well as the Database of Curated Mutations (DoCM) (Figure 4B). These genes include *PLAG1*, *CHCHD7*, *SOX17*, *TCEA1*, *NCOA2*, *TCEB1*, *HEY1*, *RUNX1T1*, *NBN*, *CNBD1*, *COX6C*, *UBR5*, *RAD21*, *EXT1*, *MYC*, *NDRG1*, *EPPK1* and *RECQL4*. Among these, *MYC* (8q24) has been studied in a variety of cancers and is known to be a cell cycle regulator, important for neoplastic transformation (36). Reuss and colleagues (37) showed that MPNST cell lines with complete *NF1* deficiency were sensitive to TRAIL-induced cell death that was critically dependent on *MYC*. Thus, targeting dysregulated *MYC* expression using BRD4 inhibitors may represent a therapeutic opportunity in NF1-associated MPNST (38). Additionally, Chr8 gains have been described in Ewing sarcoma and several pediatric soft-tissue sarcomas (39–41) and, therefore, may be a common founder event in sarcoma malignant transformation. We interrogated the expression levels of genes in this area of Chr8q using our bulk RNA-seq data. *RECQL4*, *SOX17*, *UBR5*, *MYC*, *RAD21*, and *HEY1* were the genes with the highest expression in MPNST. The expression levels of the other genes were more variable and at much lower levels, making them less likely candidates to play a role in progression of MPNST (Supplemental Figure 5). Next, observing cells with Chr8q gain compared with cells without Chr8q gain using the results of inferCNV, we obtained a list of significantly highly differentially expressed genes, which included *HEY1*, *MYC*, and *RAD21* (Figure 4C and Supplemental Table 4). However, *RECQL4*, *SOX17*, and *UBR5* were not found to be differentially expressed in the scRNA-seq data set. We next reviewed the expression of *HEY1*, *MYC*, and *RAD21* in the bulk RNA-seq data between MPNST and the PN for which there was data, and we found these 3 genes to be more highly expressed in MPNST compared with PN (Figure 4D and Supplemental Figure 6), further implicating these as candidate genes. Finally, using the cancer genome atlas (42) database, we analyzed the correlation between Chr8q gain and OS, and we found that Chr8q gain was associated with inferior OS in 2 soft-tissue sarcomas data sets (42) (Figure 5A; *P* = 0.0013, *P* = 0.0129, logrank). Additionally, using the gene expression profiling interactive analysis (GEIPA) portal available through TCGA (http://gepia.cancer-pku.cn/), we found that OS was significantly decreased in soft-tissue sarcomas with high expression of *RAD21* or *MYC*, but not for *HEY1*, *UBR5*, *RECQL4*, or *SOX17*, making *RAD21* and *MYC* the 2 most likely candidate genes (Figure 5B). As every MPNST examined in our analysis demonstrated Chr8q gain, this association may be, at least in part, accountable for the poor survival seen in MPNST patients.

## Discussion

MPNST represent a spectrum of aggressive soft-tissue sarcomas that can arise sporadically, following radiation therapy, or in the setting of NF1 (43–45). Specific therapeutic options are limited, since the pathogenesis of these tumors is still poorly understood due to their heterogeneity.

Recently, PDX models have gained traction as a preclinical platform and a tool to study tumor heterogeneity. These humanized models represent unique tools to study the characteristics of a large variety of tumor types, as early-passage PDX lines retain a high degree of similarity to the parental tumor tissues, which allows for studying the disease etiology and tumor cell origin of the human tumor (46, 47). PDX models retain both histopathologic and genetic characteristics of the tumor donor throughout several passages. Therefore, these models can be used to study drug evaluation, biomarker identification, and treatment strategies (17, 19, 20).

We have generated and characterized 8 NF1-MPNST PDX reflecting the genetic landscape and molecular heterogeneity of NF1-derived MPNST, which is, to our knowledge, the largest characterized set of NF1-MPNST PDX reported to date. This work has led to several important findings. First, we observe the vast heterogeneity of disease in a single cohort. *TP53* mutations occurred in 12.5% (1 of 8 pairs), *SUZ12* mutations in 62.5% (5 of 8 pairs), and the other 25% (2 of 8 pairs) had mutations in other cancer-related pathways, such as DNA repair genes. Second, our data show a high degree of aneuploidy in NF1-MPNST, while PN are generally diploid. This finding is important, since DNA aneuploidy is known to be an independent risk factor, in addition to histologic grade and lymphovascular invasion, for decreased metastasis-free survival in sarcomas (43). Sarcomas are, in general, more copy-number aberrant than other cancers (48), and MPNST in particular are highly copy-number aberrant (48). Our data illustrate the difference in copy-number variants between benign precursor lesions and the high-grade sarcoma after malignant transformation. Other studies of PN have shown these tumors to be diploid (20, 21). In the current study, we do detect some copy-number alterations, albeit at low levels. This difference may be due to the high depth of our sequencing or detection of some atypical cells within these PN. This cellular heterogeneity would not necessarily be unexpected, given that ANNUBP and MPNST arise within PN and these are notoriously heterogeneous tumors. Third, copy-number variation analysis highlighted a significant gain in Chr8 in NF1-MPNST, while PN did not demonstrate this finding. While previous studies show that every chromosome has been involved in numerical and/or structural aberrations in MPNST (21), 1 study reported a Chr8 gain accompanied by gains and losses in several other chromosomes in a very small sample size by array CGH (25). Our results specifically support 8q gain, an arm that encompasses several key cancer-related genes that may be important for MPNST transformation. Third, to our knowledge, we have presented the first use of scRNA-seq in MPNST, with the aim of further characterizing these clones. Our data highlight the impressive intratumoral heterogeneity within each tumor sample. However, almost every clone (>90% of cells) harbored 8q gain. In 5 of 8 PDX, Chr8q gain is seen in the trunk of the clonality tree, suggesting that it is an early copy number event. However, since it is not in the trunk of every tree, it cannot be the first copy number event in every tumor. This finding suggests that there is a preceding event that triggers the tumor to become aneuploid but that Chr8q gain likely confers a competitive advantage to the tumor and, thus, is selected early on. This hypothesis is supported by the fact that our data show gain of 8q as the most prevalent CNV event among the 74 events detected in all 8 NF1-MPNST. Additionally, we found that fluorescence in situ hybridization experiments could further substantiate Chr8 gain in PDX, parental tumors, and a validation set of other NF1-MPNST samples. These data support the notion of a selective advantage of Chr8 gain, as well, given that the percentage of cells with Chr8q gain increases in PDX compared with parental tumors. The long arm of Chr8 is the home of multiple cancer-related genes. Among these, *MYC* (8q24), has been extensively studied in a variety of cancers ; it is known to be a regulator of cell cycle and important for neoplastic transformation (36). Reuss and colleagues (37) have shown that MPNST cell lines with complete *NF1* deficiency were sensitive to TRAIL-induced cell death, and TRAIL sensitivity was critically dependent on *MYC*. Thus, *MYC* may represent a therapeutic opportunity in NF1-associated MPNST. There are several other cancer-related genes on 8q, including *HEY1* and *RAD21*, which are all candidates that we are currently evaluating. Furthermore, Chr8 gain has been described in Ewing sarcoma and other pediatric soft-tissue sarcomas (39–41) and, therefore, may be a critical event for numerous sarcomas, in addition to NF1-MPNST. In line with this, we demonstrated that Chr8 gain was associated with poor OS in the soft-tissue sarcoma data sets deposited in TCGA. Future studies will be aimed at correlation with clinical outcome in MPNST and other soft-tissue sarcoma subsets, as well as the determination of which genes on this locus are critical for MPNST progression.

In summary, we present the deep characterization of a set of 8 NF1-MPNST PDX lines. To our knowledge, our analyses included the first use of scRNA-seq in this cancer type and highlight the complex intra- and intertumoral heterogeneity of MPNST. Additionally, our data support that gain of Chr8q is a common event in MPNST pathogenesis and an area that warrants further investigation. Future studies, conducted through multiinstitutional collaboration, will be aimed at determining the strength of the correlation between CNV and clinical outcomes in a large subset of MPNST, as well as the determination of the specific genes at this locus that are critical for MPNST progression.

## Methods

### Human subjects and PDX tumor models.

Two–4 pieces of tumor tissue were collected from each surgical specimen, immediately following resection, while the tumor was being processed in pathology. Tissues were placed into DMEM during transport to the laboratory and were then used for implantation into mice, histology, and sequencing of the parental tumor. Tissue for histology was fixed in 10% formalin and stained with H&E to appropriately characterize the morphology of the implanted tumor. Tumor tissue was implanted s.c. into the flank of 5- to 6-week-old NSG mice (The Jackson Laboratory). The mean elapsed time for engraftment varied from 21 to 90 days, and engraftment occurred in 2–4 mice. Engraftment success of the PDX tumor model was defined by the ability for the line to be serially transplanted. Passage 5 was used for comparison studies.

### Histological evaluation of primary and PDX tumors.

Sections (6 μm) were prepared from formalin-fixed paraffin-embedded blocks for IHC stains with adequate controls. IHC was performed using the Avidin/Biotin blocking kit (Vector Labs, SP-2001) staining with antibodies against S100 (1:250 dilution; catalog ab41548, Abcam), KI-67 (prediluted; catalog 790-4286, Ventana), and COL4A (1:1000 dilution; catalog ab6586, Abcam) as previously described (49). Immunolabeling for anti-histone H3 (tri methyl K27) (1:200 dilution; catalog ab6002, Abcam) was performed on formalin-fixed, paraffin embedded sections. Briefly, following dewaxing and rehydration, slides were immersed in 1% tween-20; then, heat-induced antigen retrieval was performed in a steamer using 1.0 mM EDTA pH 9.0 (catalog AP9004500, Thermo Fisher Scientific) for 45 minutes. Slides were rinsed in PBST, and endogenous peroxidase and phosphatase was blocked (catalog S2003, Dako). Sections were then incubated with primary antibody anti–histone H3 (tri methyl K27) (1:200 dilution; catalog ab6002, Abcam) for overnight at 4°C. The primary antibodies were detected by 30-minute incubation with HRP-labeled secondary antibody (catalog PV6114, Leica Microsystems), followed by detection with 3,3′-Diaminobenzidine (Sigma-Aldrich), counterstaining with Harris hematoxylin, dehydration, and mounting.

### WES, WGS, and RNA-seq library construction and sequencing.

Each tumor and xenograft had 2 enriched libraries constructed (*n* = 16), and the normal germline samples had a single enriched library constructed (*n* = 8). Exome libraries were captured with an IDT exome reagent and were then pooled with a WGS library for sequencing on an Illumina HiSeq4000 with at least 1000× coverage for WES and 30× coverage for WGS. RNA was prepared with a TrueSeq stranded total RNA library kit and were then sequenced on an Illumina HISeq4000 at an average of 52M reads/sample. All WES/WGS/RNA-seq samples were trimmed and quality controlled via Trim Galore (https://www.bioinformatics.babraham.ac.uk/projects/trim_galore/).

### WES data analysis.

WES and WGS data were aligned against reference sequence hg38 via BWA-MEM (50) with base quality score recalibration (BQSR). For PDX sequence data, Disambiguate v1.0 (51) was used to filter out mouse-derived reads using mouse (GRCm38.p6, GENCODE release M19) and human (hg38) reference genomes, and the resulting reads were deduplicated. Structural variants (SVs) and large indels were detected using Manta (52). SNVs and small indels were detected using VarScan2 (53), Strelka2 (54), MuTect2 (55), and Pindel (56) via the somatic pipelines available at https://github.com/genome/analysis-workflows (CommitID: 799e68848a4b8265e7b28d37c718a6ba7212aeeb; commitURL: https://github.com/genome/analysis-workflows/commit/799e68848a4b8265e7b28d37c718a6ba7212aeeb), which includes best-practices variant filtering and annotation with Variant Effect Predictor (VEP; version 95) (57). Manual review was used to remove additional sequencing artifacts. Germline and somatic variants reported by the variant-detection pipeline were compared, and any intersecting variants were removed from the somatic variant gene list, thus filtering out the germline variants. Common variants found in the 1000 Genomes MAF > 0.05 were filtered out. Waterfall somatic variant plots were created with GenVisR (58) by including somatic variants that occurred in each area.

### Bulk RNA-seq analysis.

Aligned RNA reads were aligned to their respective genome assembly. Initial WU-225, WU-356, WU-368, WU-386, WU-436 primary tumor, and PDX samples were aligned to GRCh37. The latest JH 2-002, JH 2-023, and JH 2-031 were aligned to GRCh38. Mouse-derived reads were filtered using Xenosplit (59). RNA reads were quantified by using the expectation-maximization (EM) quantification on PartekFlow (60). Gene counts and transcript counts were normalized by using the DESeq2 (61) package. PartekFlow was used for heatmap visualizations (59–61).

### Inference of copy number variations on WGS data.

CNVkit was used to infer and visualize copy number from high-throughput WGS data. Coverage for each bait position in the exome reagent was calculated; then, segments of constant copy number were identified using circular binary segmentation. Data were plotted to provide visualization of CNVs. John Hopkin’s CNV data from PN was directly imported from Synapse (https://www.synapse.org/#!Synapse:syn18634452).

### FISH.

Interphase FISH was performed on formalin-fixed paraffin-embedded tissue sections cut at a thickness of 5 μm on positively charged microscope slides. The paraffin was removed from the sections with 3 washes of 5 minutes each in CitriSolv (catalog 04355121, Thermo Fisher); pepsin (ThermoFisher). The slides were then hydrated in 2 washes of absolute ethanol for 1 minute each and allowed to air dry. The slides were processed through a pretreatment solution of sodium thiocyanate, which had been preheated to 80°C. After a 3-minute wash in distilled water, the tissue was digested in protease solution (pepsin in 0.2N HCl) for 15 minutes at 37°C, followed by another 3-minute wash in distilled water. The slides were allowed to air dry, after which they were dehydrated by passing through consecutive 70%, 85%, and 100% ethanol solutions for 1 minute each. The slides were again allowed to air dry before applying prepared probe mixture. Probes used were a centromeric-enumerating probe Vysis CEP 8 (D8Z2) SpectrumGreen combined with locus-specific probe Vysis LSI MYC SpectrumOrange (Abbott Molecular). Probes were diluted at a concentration of 1:50 in *t*DenHyb-2 hybridization buffer (Insitus Biotechnologies Inc.) and well mixed. Next, diluted probe was applied to the appropriate slide to cover the tissue section, and the section was cover slipped. Codenaturation was achieved by incubating the slides at 73°C for 5 minutes in a slide moat. Hybridization occurred by transferring the slides to a 37°C slide light-shielded, humid slide moat overnight. After hybridization, the coverslips were removed and the slides were immersed in 75°C wash solution (2× SSC/0.3% NP-40) for 2 minutes, followed by a 1-minute wash in a jar containing the same solution at room temperature. The slides were allowed to air dry in the dark and were then counterstained with 10 μL of DAPI II (Abbott Molecular Inc.). Slides were examined using an Olympus BX61 fluorescent microscope with appropriate filters for SpectrumOrange, SpectrumGreen, and the DAPI counterstain. The signal patterns were documented using a JAI Progressive Scan camera and CytoVision Imaging System.

### scRNA-seq.

scRNA-seq was performed on the Chromium controller (10X Genomics) and sequenced using Illumina NextSeq sequencer. MPNST PDXs were minced in a cell culture dish and immediately dissociated in tumor dissociation media (DMEM supplemented with 10% FBS, 100 U/mL penicillin/streptomycin solution, dispase II, collagenase I and DNase I) on a gentleMACS dissociator (Miltenyi Biotec). Single-cell suspension was counted using both Cellometer Auto 2000 fluorescent viability cell counter (Nexcelom) and Luna-FL dual fluorescent cell counter (Logos Biosystems). The manufacturer’s protocol was followed with a target capture of 6000 live cells from each sample. WU-386, WU-225, WU-368, JH 2-023, JH 2-002, WU-356, and WU-436 were processed using the 3′ Library and Gel Bead Kit v2 (10X Genomics). PDX JH 2-031 was processed using the 3′ Library and Gel Bead Kit v3 (10X Genomics). Each sample was processed on an independent Chromium Single Cell A Chip. Cell partitioning was completed successfully with uniform emulsion consistency and was followed with reverse transcription in a thermal cycler. All subsequent steps of library preparation and quality control were performed as described in the 10X Genomics 3′ Single cell User Guide. Gene expression libraries were sequenced using the NextSeq High Output 150-cycle v2.5 Flow Cell.

### scRNA-seq data analysis.

The standard 10X Genomics Cell Ranger (version 3.1.0) pipeline was used to extract FASTQ files and to perform data processing. Sequenced reads were aligned to the 10X Genomics provided human and mouse reference (refdata-cellranger-GRCh38-and- mm10-3.1.0). Human genome aligned reads were used in the downstream analysis. Each sample was initially processed separately and then integrated. For individual tumor analysis, “filtered_feature_bc_matrix” was imported using the Read10X function in Seurat (version 3.0) and a Seurat object was created keeping all genes expressed in more than 4 cells and all cells with more than 200 genes detected. Cell were further filtered after an initial quality control step in Seurat to retain cells with unique gene counts that were between 200 and 6000 and the percentage of mitochondrial genes that was lower than 25%. After removing the unwanted cells, the data were normalized using the “LogNormalize” method with a scale factor of 10,000 and scaled to remove the unwanted sources of variation from mitochondrial gene content and number of detected unique molecules in each cell. The top 2000 variable genes were calculated using the FindVariableFeatures function in Seurat and used in the following principal component analysis (PCA) to reduce dimensionality of the data. The first 40 PCs were used to find clusters in the data with the resolution set to 1.2. For the integration of data obtained from all PDX, all 10X Genomics Cell Ranger processed, human hg38 genome annotated count matrices (1 from each PDX) were imported into Seurat and integrated into 1 data set following the standard integration pipeline. The top 2000 variably expressed genes of each PDX were used in the anchor-based integration with the first 30 PCs, and the same 2000 most variable genes were used in the PCA to reduce dimensionality of the data. The first thirty PCs were used with the FindClusters function with the resolution set to 0.5 to find distinct clusters. We used Uniform Manifold Approximation and Projection (UMAP) as the dimension reduction technique here to visualize the distinct clusters detected among the 8 PDXs. A total of 14 clusters was detected in the integrated data, and each of these clusters represents a group of cells with a unique transcriptional profile in comparison with the other cells in the data. The FindAllMarkers function was used to identify conserved cell markers when comparing one cell cluster to the other clusters altogether with the following parameters: only.pos = TRUE, min.pct = 0.25, logfc.threshold = 0.25. The function CellCycleScoring was used to calculate the cell cycle score of each cell and to add the annotation of cell cycle state that was scored the highest. Seurat (version 3.0) was used for the analysis in R (version 3.6.0) and data visualization.

### Inference of copy number variations and clonality analysis.

Copy number variations of each PDX were inferred from scRNA-seq using the R package inferCNV (https://github.com/broadinstitute/infercnv). Raw counts of each PDX were extracted from the Seurat object of individual samples following the recommendations in the “Using 10x data” section of inferCNV. A scRNA-seq data set of PN that is composed of 19,000 cells randomly selected from 4 human primary PNs was used to provide normal reference cells. The following parameters were used for the inferCNV analysis: cutoff = 0.1 to use the genes with a mean number of counts larger than 0.1, denoise = T to obtain the residual CNV signal by subtracting the mean CNV signal from the normal cells to, and HMM = T to apply the default Hidden Markov Model. To rule out false-positive CNVs detected by the Hidden Markov Model, a Bayesian latent mixture model was implemented to identify the posterior probabilities of alteration status in each cell with each gene or CNA regions. The default probability threshold of 0.5 was used. To determine the clonality within each tumor due to the intratumoral heterogeneity, the “subcluster” analysis mode was used with the tumor_subcluster_partition_method set to random_trees and tumor_subcluster_pval set to 0.05. A 6-state CNV model was adapted by inferCNV, and each detected CNV was predicted to be in one of the following CNV levels: State 1, complete loss; State 2, loss of 1 copy; State 3, neutral; State 4, addition of 1 copy; State 5, addition of 2 copies; and State 6 addition of more than 2 copies. The CNV was considered as “loss” for States 1 and 2 or “gain” for States 4, 5, and 6 events. Each CNV events detected was summarized in large-scale genomic regions and attributed to the chromosome arm levels according to the hg38 cytoband information (http://hgdownload.cse.ucsc.edu/goldenpath/hg38/database/). The identified CNV types and calculated percentage of cells within each CNV type were plotted as a phylogenetic tree. The Uphyloplot2 (https://github.com/harbourlab/UPhyloplot2) was used to visualize the clonality determined by inferCNV analysis of each PDX. CNV data were processed in R (version 3.6.2), and the summary of each tumor is presented in Supplemental Table 4. All detected CNV events were summarized and the percentage of cells exhibiting each characteristic CNV event in each PDX was plotted in a heatmap using R package (ComplexHeatmap). A range of CNV features was extracted from the inferCNV result and added to the Seurat metadata for UMAP visualization using the function add_to_seurat. The function FindMarkers was used to derive differentially expressed genes between cells that do exhibit gain of Chr8q and cells that do not. These genes are provided in Supplemental Table 5.

### Data storage and sharing.

All shared data are stored in the SYNAPSE repository (doi:10.7303/syn11638893) and will be made available to all researchers.

### Statistics.

OS in STS patients with Chr8 gain versus no gain was estimated with Kaplan-Meier analysis, and differences between the cohorts was assessed with log-rank test and the Cox proportional hazards model, which was used to calculate the hazard ratio (HR) and its associated 95% CI using the R package “survival”. OS based on high versus low expression of each of the candidate genes was estimated with Kaplan-Meier analysis, and differences between the cohorts was assessed with the Cox proportional hazards model, which was used to calculate the HR and its associated 95% CI on the GEIPA website (http://gepia.cancer-pku.cn/). Analyses were considered statistically significant if *P* < 0.05. Coefficient of determination (or *R^2^* value) for RNA-seq expression plots and VAF plots were calculated using Spearman correlation and Pearson correlation, respectively, using the R built-in function.

### Study approval.

The present studies in humans were reviewed and approved by IRBs at Washington University in St. Louis (protocol no. 201203042) and Johns Hopkins University (protocol no. J1649). All subjects provided written informed consent prior to participation in the study.

## Author contributions

ACH obtained funding, conceptualized the research, designed and analyzed experiments, and edited the manuscript. JFS, CAP, and SL aided in research conceptualization and experimental design and edited the manuscript. LD aided in experimental design and edited the manuscript. CD, CIM, Xiyuan Zhang, and ZZ are co–first authors that contributed equally and are listed in alphabetical order; they designed experiments, analyzed data, and wrote the manuscript. Xiaochun Zhang, KP, and TP generated PDX and edited the manuscript. CM, HX, XW, KY, JM, SJCG, YW, AG, PAJ, SD, FJR, and HB performed experiments, analyzed data, and edited the manuscript.

## Supplementary Material

Supplemental data

## Figures and Tables

**Table 1:**
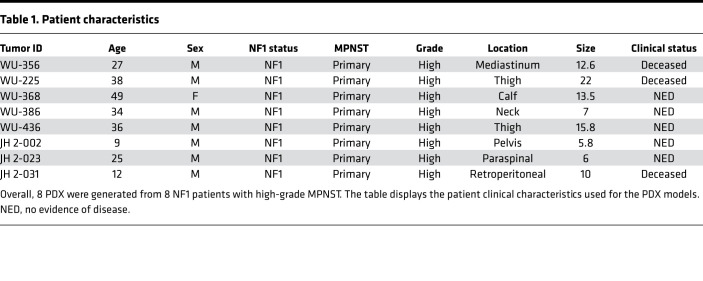
Patient characteristics

**Table 2:**
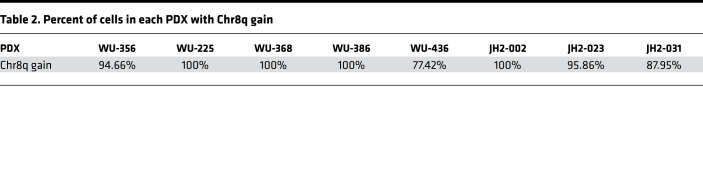
Percent of cells in each PDX with Chr8q gain

**Table 3:**
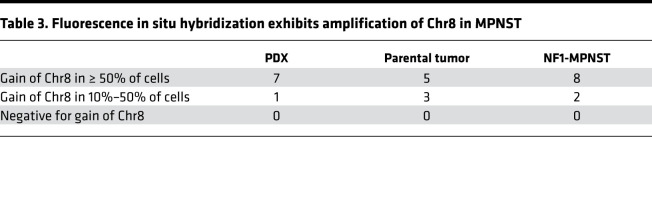
Fluorescence in situ hybridization exhibits amplification of Chr8 in MPNST

## Version 1 02/16/2021

In-Press Preview

## Version 2 03/22/2021

Electronic publication
