# Supplementary material for: Chromosome 8 gain is associated with high-grade transformation in MPNST
Source: JCI Insight. 2021 Mar 22;6(6):e146351. doi: 10.1172/jci.insight.146351 (PMC8026192; doi:10.1172/jci.insight.146351)
Supplement: Supplemental data [file jciinsight-6-146351-s167.pdf]

Supplementary data

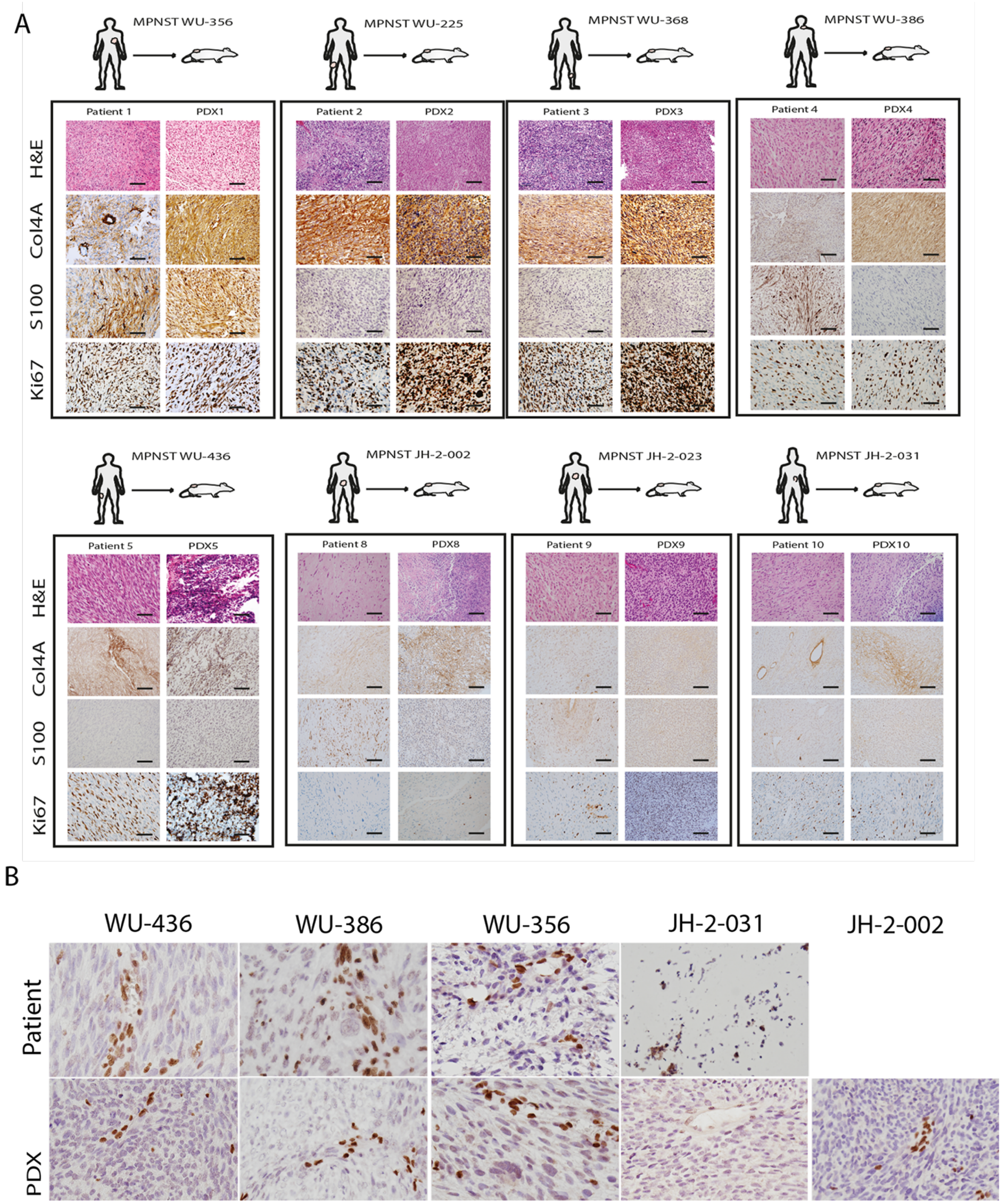

Supplemental Fig. 1: **Histological Comparison of tumor-of-origin to patient-derived-xenograft (PDX).**

(A) Immunoreactivity for H&E, Col4A, S100, and Ki67 in patient tumors of origin and corresponding PDX tumors with schematic depicting the site of the original tumor in the patient. Scale bar = 20um. (B) Patient and PDX tumors show loss of H3K27me3 by immunohistochemistry. Further patient tissue is unavailable for JH 2-002.

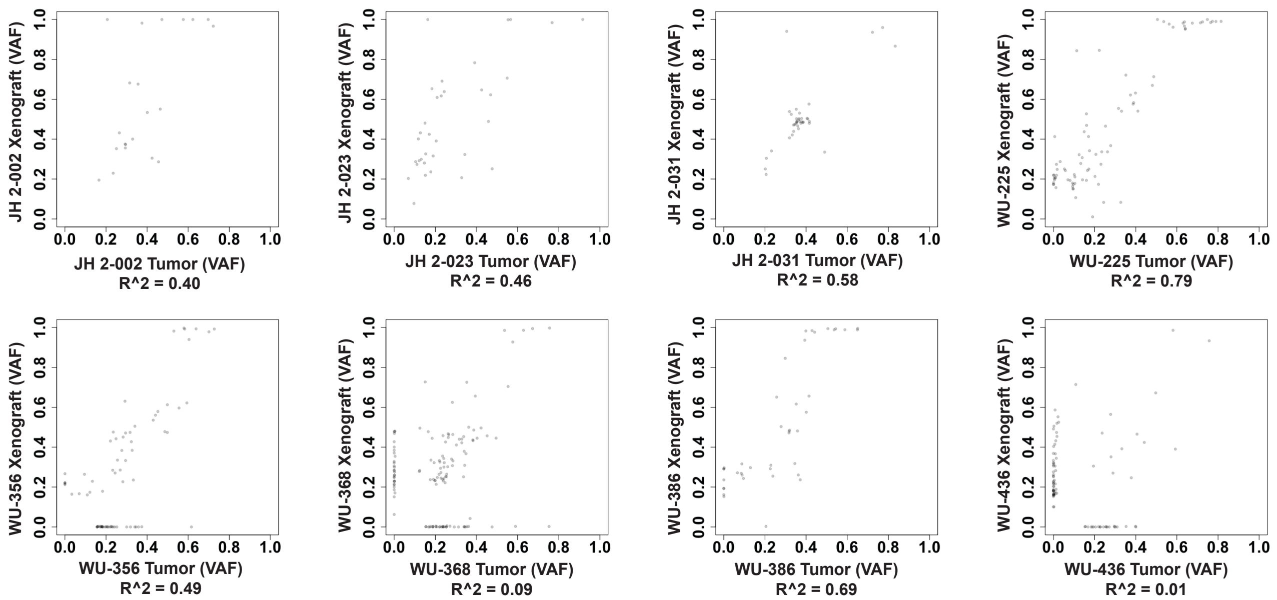

Supplemental Fig. 2: Scatter plots displaying the correlation between tumor of origin and PDX DNA.
Tumor of origin versus patient derived xenograft (PDX) variant allele frequency (VAF) plots. Variance is in relation to the identity line, therefore, points along axis reflect evidence of heterogeneity. The correlation coefficients (on a scale of 0 to 1) are included underneath. Coefficient of determination (or R-square value) for variant allele frequency (VAF) plots were calculated using Pearson correlation using the R built-in function.

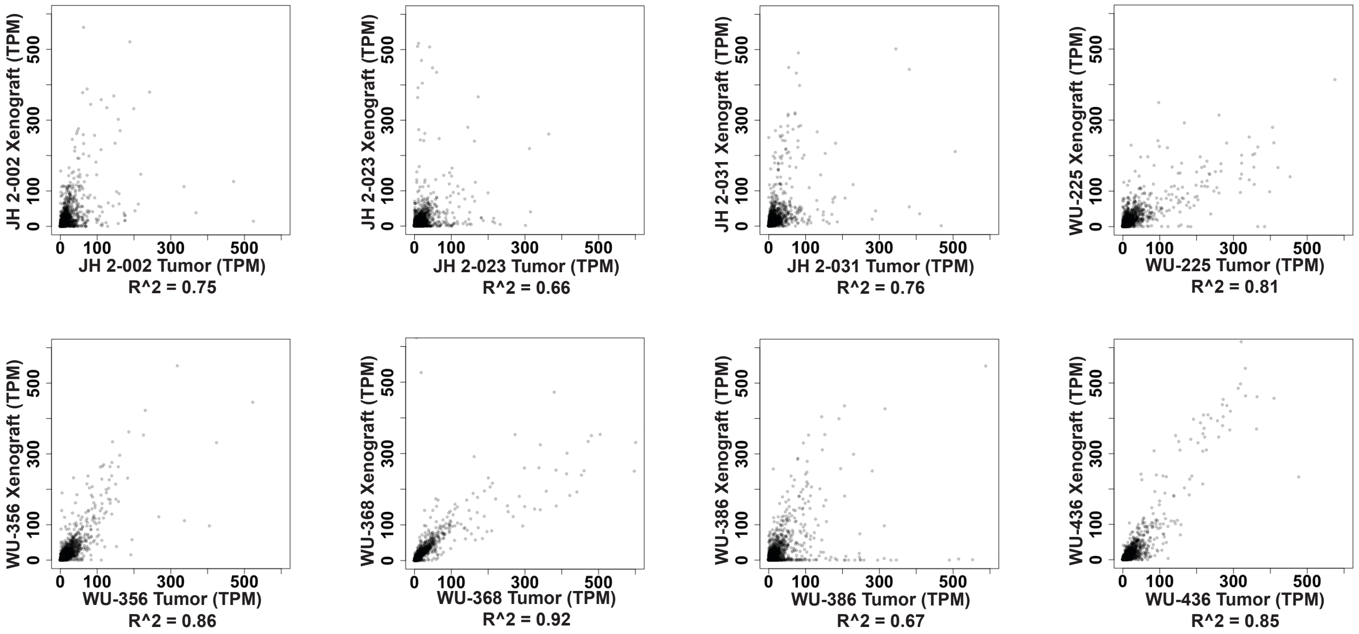

Supplemental Fig. 3: Scatter plots displaying the comparison of RNAseq data between tumor of origin and PDX RNAseq.

Simple X/Y scatterplots showing normalized counts gene expression levels of each tumor compared to its respective xenograft. Variance is in relation to the identity line, therefore, points along axis reflect evidence of heterogeneity. The correlation coefficients (on a scale of 0 to 1) are included underneath. Coefficient of determination (or R-square value) for RNA-seq expression plots were calculated using Spearman correlation using the R built-in function.

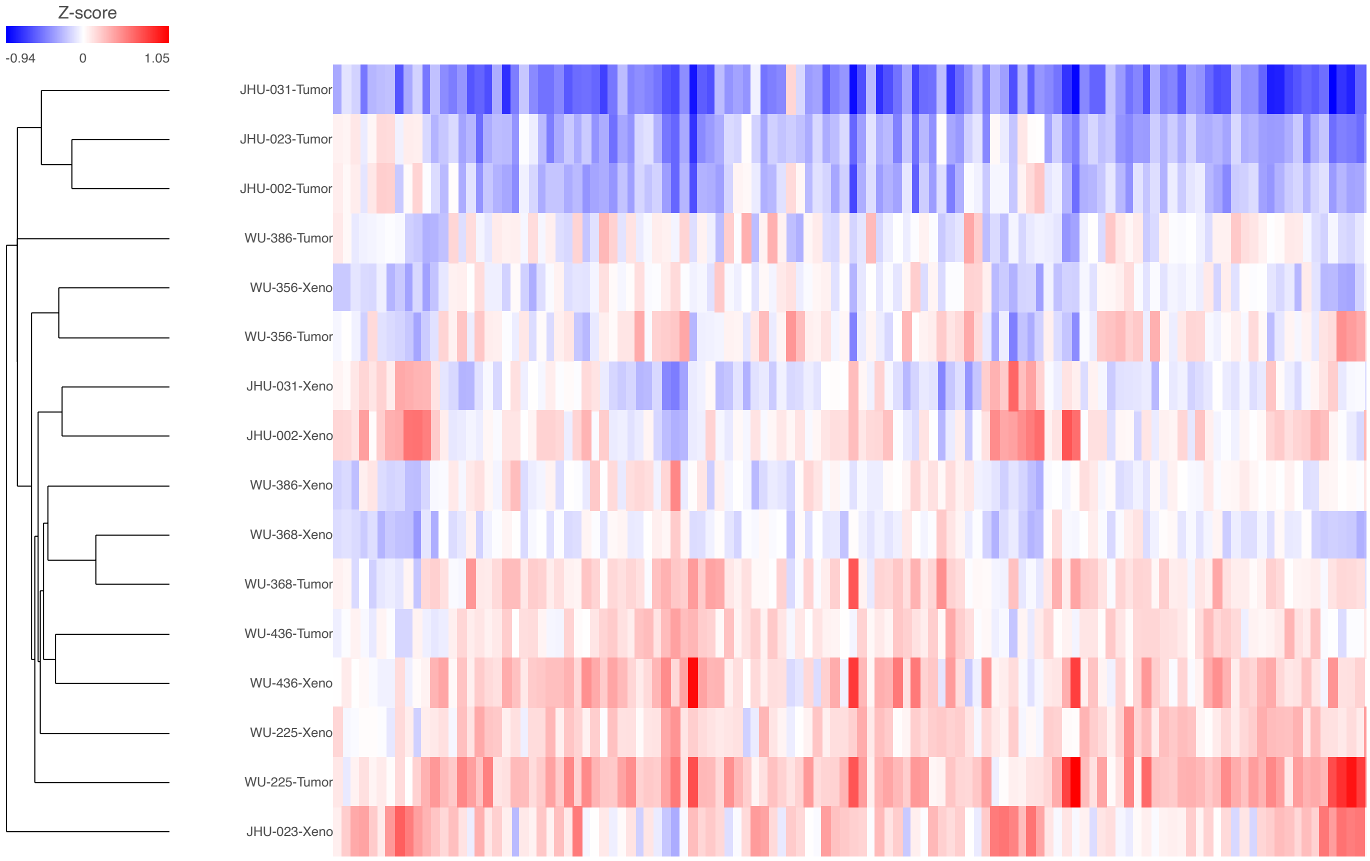

Supplemental Fig. 4:
Heatmap of PDX and parental tumor samples with list of all genes captured by bulk RNA-seq. The row represents the sample name and the column represents all genes found in bulk RNA-Seq.

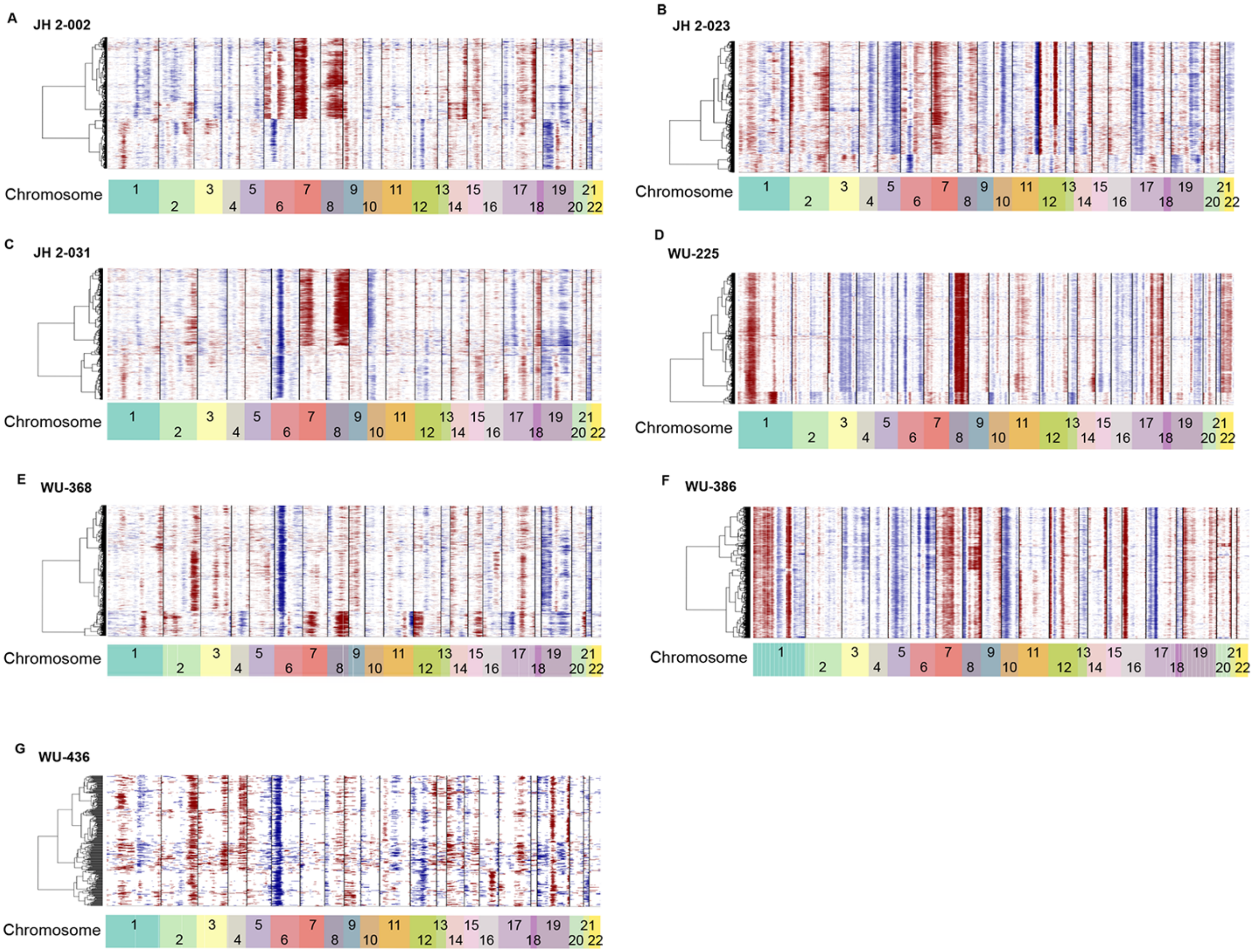

Supplemental Figure 5: Gain of chromosome 8 is common in MPNST PDX.
A-G) copy number variation (CNV) heatmap with hierarchical clustering of each MPNST PDX showing results of the inferCNV analysis.

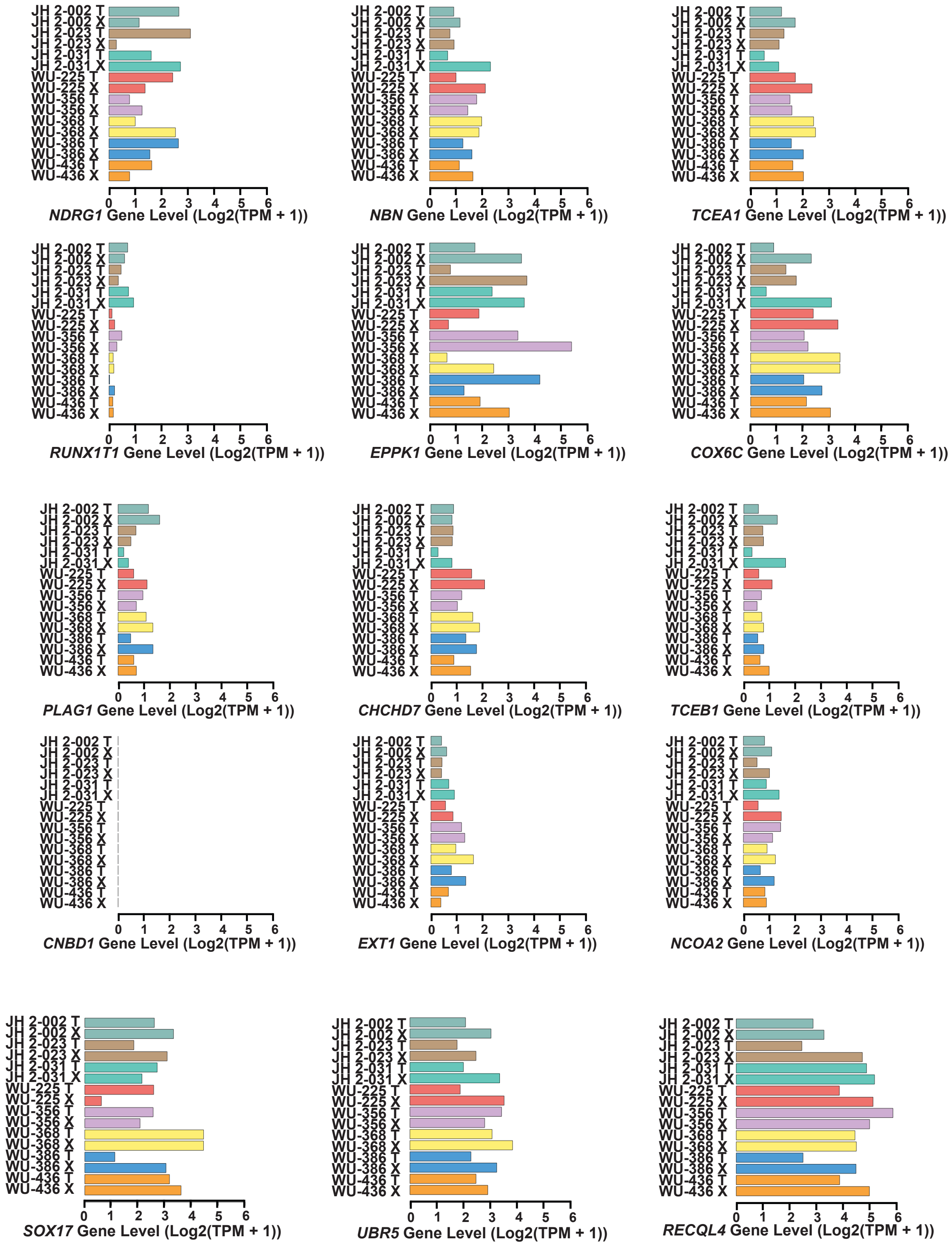

Supplemental Fig. 6: Expression of additional cancer-related genes on Chromosome 8

The barplots represent the RNA expression of additional cancer-related genes on chromosome 8 in our cohort.

Tables

PDX ID	Total Cells Captured	Mean reads per cell	Median genes per cell	Cell count-GRCh38
WU-386	3192	93371	4061	3106
WU-225	4976	99624	5451	4948
WU-368	5268	125664	976	1558
WU-356	1968	114580	784	1644
WU-436	2249	1265194	670	316
JH2-023	4026	32401	2134	4590
JH2-002	2390	75224	1812	2279
JH2-031	6582	89513	1255	5614

Supplemental Table 1: Single-cell RNA sequencing QC

	JH2-002	JH2-023	JH2-031	WU-225	WU-356	WU-368	WU-386	WU-436
0	0.26853883	0.28540305	0.15817599	0.40602264	0.25364964	0.05455712	0.42691565	0.29113924
1	0.12856516	0.19978214	0.19059494	0.02081649	0.20194647	0.02759949	0.01835158	0.23101266
2	0.04651163	0.12636166	0.01531885	0.21059014	0.0729927	0.02053915	0.17611075	0.08227848
3	0.08951294	0.18126362	0.12771642	0.00181892	0.15632603	0.06931964	0.00128783	0.08544304
4	0.1667398	0.05141612	0.18453865	0	0.1107056	0.15789474	0	0.08227848
5	0.01140851	0.00457516	0.00552191	0.20230396	0.00790754	0.00449294	0.21796523	0.01265823
6	0.06537955	0.04662309	0.08656929	0.00080841	0.05778589	0.48395379	0.00450741	0.10126582
7	0.02281703	0.04901961	0.01086569	0.10448666	0.03832117	0.01797176	0.08757244	0.0443038
8	0.05221588	0.01176471	0.07908799	0.05234438	0.06812652	0.00577664	0.06535737	0
9	0.11057481	0.02287582	0.04453153	0	0.01946472	0.0012837	0	0.00632911
10	0.02062308	0	0.03758461	0	0	0.05840822	0	0.00949367
11	0.00482668	0.01132898	0.04061275	0.0004042	0.00182482	0.01412067	0.00032196	0.03164557
12	0.00263273	0.00326797	0.0122907	0.0004042	0.00121655	0.0661104	0.00160979	0.01265823
13	0.00965336	0.00631808	0.00659067	0	0.00973236	0.01797176	0	0.00949367

Supplemental Table 2: Distribution of cell clusters among each PDX

	WU-356	WU-225	WU-368	WU-386	WU-436	JH 2-002	JH 2-023	JH 2-031
**8q gain**	94.6570397	100	100	100	77.4193548	100	95.8634953	87.9599306
**14q gain**	30.4693141	99.9595469	100	100	89.0322581	100	100	93.0263918
**17q gain**	21.1552347	100	100	100	100	100	100	61.452514
**1q gain**	100	100	100	100	72.9032258	53.5601764	87.745605	54.4789058
**6p loss**	100	100	99.9354839	100	100	27.4102079	82.9886246	97.3030245
**2q gain**	80.433213	90.6148867	79.9354839	43.0560052	100	60.3024575	100	100
**19q gain**	88.8086643	90.5744337	51.483871	100	97.7419355	95.5891619	79.9120993	39.7418609
**1p gain**	36.534296	100	24.7741935	100	89.0322581	38.1222432	100	86.630707
**12q gain**	79.3501805	100	20	100	45.483871	79.7731569	100	48.2180697
**15q gain**	7.6534296	46.6019417	93.8064516	69.9579152	50.6451613	100	52.9472596	55.9815065
**9q gain**	0	90.5744337	80	100	27.4193548	89.0989288	13.081696	78.2893469
**5q loss**	1.37184116	100	55.2903226	100	53.8709677	73.7870195	100	53.8624542
**20q gain**	71.1913357	100	20	100	0	24.1965974	95.8634953	92.1017145
**7p gain**	56.7509025	100	20.0645161	100	0	64.4612476	91.0548087	100
**3q loss**	68.1588448	99.9595469	20	100	0	100	35.9358842	45.5210942
**7q gain**	79.3501805	9.42556634	69.1612903	100	0	64.4612476	86.1685626	58.8903872
**2p gain**	91.8411552	100	26.1290323	100	0	20.2268431	100	35.5422847
**11p gain**	19.2779783	90.6148867	49.0967742	100	65.1612903	22.9363579	0	64.7466769
**8p loss**	0	99.9595469	73.8064516	100	5.16129032	0	100	20.7281834
**1q loss**	0	100	0	100	45.483871	61.8777568	86.918304	13.369293
**13q loss**	5.34296029	99.9595469	35.4193548	100	2.25806452	0	100	6.97360817
**12p gain**	79.3501805	0.04045307	26.1935484	100	0	0	100	50.9921017
**19q loss**	17.9061372	100	48.4516129	0	21.6129032	73.7870195	100	79.2718166
**11q loss**	76.534296	99.9595469	20.0645161	43.0560052	23.5483871	24.7637051	94.131334	0
**21q loss**	5.34296029	90.5744337	99.9354839	0	27.4193548	35.5387524	60.8841779	32.7682527
**17q loss**	59.7833935	100	6.90322581	100	0	11.9092628	3.8262668	57.5611635

**5p gain**	13.9350181	90.5744337	31.6774194	100	46.4516129	0	92.78697	15.3149682
**4q loss**	79.3501805	99.9595469	20	91.9391389	0	60.0504096	86.1685626	0
**16p gain**	79.3501805	0.04045307	20	100	0	10.9010712	86.1685626	6.97360817
**17p loss**	0	90.6148867	13.1612903	100	0	61.8777568	89.9948294	13.369293
**6q loss**	0	99.9595469	0	100	0	0	0	20.7281834
**4q gain**	5.34296029	0.04045307	55.2903226	0	85.8064516	38.1222432	13.081696	93.0263918
**3q gain**	31.8411552	49.1100324	93.0967742	0	94.8387097	11.9092628	22.9317477	33.7507224
**10q loss**	0	19.7006473	0	100	0	77.189666	92.78697	20.7281834
**19p loss**	21.1552347	0	86.9032258	0	72.9032258	61.0586011	13.8314374	54.4789058
**16q gain**	5.34296029	0	22.3225806	97.6367757	27.4193548	9.3257719	89.2450879	8.34136005
**19p gain**	0	13.6731392	0	100	34.516129	0	96.9234747	6.97360817
**10p loss**	68.1588448	89.7249191	0	27.5493687	0	10.9010712	3.07652534	58.8903872
**1p loss**	51.6245487	0	0	100	0	0	86.1685626	0
**12q loss**	6.71480144	7.36245955	0.06451613	0	78.3870968	38.1222432	70.7342296	28.2797149
**12p loss**	0	99.9595469	0	100	26.1290323	15.5009452	5.86866598	20.7281834
**22q gain**	0	99.9595469	0	97.6367757	0	0	0	6.97360817
**3p loss**	11.1913357	59.1423948	0.06451613	75.4613143	0	64.4612476	47.8024819	0
**9q loss**	8.15884477	76.0113269	0	71.090968	0	0	89.4002068	0
**10q gain**	59.7833935	92.6375405	0.06451613	0	4.19354839	22.9363579	0	58.8903872
**2q loss**	5.34296029	100	0	39.4626093	0	46.4398236	30.8428128	12.0400694
**15q loss**	30.7581227	97.9773463	0	100	5.16129032	0	0	0
**11q gain**	0	21.9660194	26.1290323	54.5807705	0	15.5009452	39.1158221	0
**5q gain**	4.40433213	9.38511327	55.2903226	0	13.2258065	11.9092628	3.07652534	25.4093624
**6p gain**	0	9.42556634	20.0645161	0	0	72.5897921	96.9234747	0
**6q gain**	11.1913357	0.04045307	0.06451613	0	0	64.4612476	76.9906929	51.916779
**16q loss**	31.8411552	100	6.19354839	2.36322434	51.9354839	2.58349086	7.21302999	0

**8p gain**	79.3501805	99.9595469	0.06451613	0	0	10.9010712	0	13.369293
**18q gain**	0	90.6148867	0.06451613	0	0	0	0	100
**18q loss**	0	9.38511327	0	100	0	0	17.0113754	0
**21q gain**	11.1913357	0.04045307	0	100	0	0	0	13.369293
**9p gain**	5.34296029	0	20.0645161	0	68.3870968	61.8777568	7.21302999	0
**17p gain**	13.9350181	7.36245955	0	0	5.16129032	28.7964713	5.86866598	39.7418609
**14q loss**	19.566787	0.04045307	6.90322581	0	0	0	22.1044467	6.97360817
**3p gain**	0	80.3398058	26.8387097	0	27.0967742	0	3.07652534	0
**11p loss**	0	7.40291262	0	0	2.25806452	11.9092628	100	0
**20p gain**	0	9.38511327	0.06451613	67.2709615	0	9.07372401	22.1044467	0
**16p loss**	0	85.3964401	0	0	0	0	7.21302999	0
**20p loss**	0	90.5744337	0	5.69763678	0	0	0	0
**22q loss**	0	10.315534	6.12903226	0	2.25806452	0	64.0641158	0
**2p loss**	0	13.7135922	0	0	0	0	0	18.0312079
**7p loss**	0	0	22.3225806	0	4.19354839	0	0	0
**4p gain**	0	0.04045307	13.1612903	0	2.25806452	0	9.85005171	0
**13q gain**	11.1913357	0.04045307	0	0	0	0	0	13.369293
**10p gain**	0	0	0	0	0	0	17.0113754	0
**4p loss**	0	9.38511327	0	0	0	0	0	0
**18p gain**	0	2.02265372	0	0	0	0	0	13.369293
**20q loss**	0	0	0.06451613	5.69763678	4.19354839	0	0	0
**18p loss**	0	0	0	0	0	0	0	0

Supplemental Table 3: Detailed information of clonality calculation from inferCNV

	genes	p_val	avg_logFC	pct.1	pct.2	p_val_adj	chr
137	*CD63*	0	1.56849917	0.853	0.53	0	chr12
180	*COL6A1*	0	1.60851759	0.81	0.235	0	chr21
206	*CTHRC1*	0	1.78973995	0.837	0.313	0	chr8
237	*DLK1*	0	1.98257785	0.778	0.163	0	chr14
251	*EEF1D*	0	0.98536258	0.918	0.641	0	chr8
365	*IGF2*	0	2.54555309	0.578	0.005	0	chr11
503	*NEAT1*	0	1.82845077	0.862	0.383	0	chr11
523	*NUPR1*	0	1.23828515	0.727	0.089	0	chr16
71	*B2M*	5.65E-306	1.35108218	0.89	0.58	1.66E-301	chr15
779	*TIMP1*	4.27E-305	1.38515144	0.855	0.468	1.25E-300	chrX
772	*TAGLN2*	4.39E-293	1.17018998	0.702	0.179	1.29E-288	chr1
493	*NDUFA4*	2.10E-290	0.99344943	0.867	0.505	6.15E-286	chr7
323	*GNB2L1*	4.74E-287	2.36400984	0.53	0.001	1.39E-282	chr5
399	*LDHA*	6.80E-287	1.07809843	0.875	0.556	2.00E-282	chr11
51	*ATP5E*	1.23E-286	1.74022421	0.53	0.001	3.59E-282	chr20
60	*ATP5L*	2.21E-286	1.39552167	0.529	0.001	6.50E-282	chr11
91	*C14orf2*	2.51E-286	1.48228773	0.529	0.001	7.38E-282	chr14
775	*TCEB2*	3.28E-286	1.66695322	0.529	0.001	9.62E-282	chr16
48	*ATP5B*	6.01E-286	1.36937571	0.528	0.001	1.76E-281	chr12
66	*ATPIF1*	6.55E-284	1.23247632	0.526	0.001	1.92E-279	chr1
710	*SHFM1*	1.74E-283	1.11380098	0.526	0.001	5.11E-279	chr7
55	*ATP5G3*	4.94E-283	1.23213217	0.524	0.001	1.45E-278	chr2
58	*ATP5J*	5.22E-283	1.03121765	0.524	0.001	1.53E-278	chr21
328	*GPX1*	3.39E-282	1.19178617	0.524	0.001	9.94E-278	chr3
61	*ATP5O*	4.76E-282	1.03948308	0.523	0.001	1.40E-277	chr21
570	*PLD3*	6.51E-281	1.08527458	0.767	0.299	1.91E-276	chr19
200	*CRIP2*	2.83E-280	0.72571445	0.607	0.056	8.32E-276	chr14
774	*TCEB1*	4.38E-280	1.10188505	0.521	0.001	1.29E-275	chr8
576	*PLP2*	7.84E-279	0.71195934	0.627	0.084	2.30E-274	chrX
47	*ATP5A1*	1.25E-278	1.18747793	0.519	0.001	3.67E-274	chr18
54	*ATP5G2*	1.01E-276	0.98864548	0.516	0.001	2.96E-272	chr12
50	*ATP5D*	1.07E-276	0.91859071	0.516	0.001	3.14E-272	chr19
90	*C14orf166*	3.06E-275	0.89135035	0.514	0.001	8.98E-271	chr14
98	*C19orf43*	1.59E-273	0.77616357	0.511	0.001	4.68E-269	chr19
56	*ATP5H*	2.44E-272	0.85641502	0.51	0.001	7.17E-268	chr17
588	*POSTN*	2.38E-271	1.38430018	0.7	0.14	6.97E-267	chr13
132	*CD151*	4.38E-269	0.73289773	0.697	0.154	1.29E-264	chr11

856	*WBP5*	5.35E-269	0.91818938	0.505	0.001	1.57E-264	chrX
505	*NGFRAP1*	9.18E-269	0.90854731	0.505	0.001	2.69E-264	chrX
136	*CD59*	1.20E-268	0.8403912	0.659	0.139	3.52E-264	chr11
656	*RP11-14N7.2*	2.65E-268	0.89827715	0.503	0	7.77E-264	chr1
59	*ATP5J2*	2.33E-267	0.83737858	0.503	0.001	6.83E-263	chr7
349	*HN1*	2.37E-267	1.27019371	0.503	0.001	6.95E-263	chr17
510	*NPC2*	1.67E-266	1.08450873	0.761	0.291	4.91E-262	chr14
343	*HLA-B*	7.41E-266	1.27261371	0.846	0.431	2.17E-261	chr6
53	*ATP5G1*	1.97E-265	1.07347016	0.5	0.001	5.77E-261	chr17
476	*MYEOV2*	5.45E-265	0.70712602	0.499	0	1.60E-260	chr2
582	*POLR2J3*	8.57E-264	0.59523707	0.529	0.016	2.52E-259	chr7
737	*SNHG7*	2.59E-262	0.71114696	0.736	0.195	7.60E-258	chr9
57	*ATP5I*	2.63E-262	0.79199311	0.496	0.001	7.70E-258	chr4
361	*IER3*	1.06E-261	0.92288238	0.636	0.095	3.12E-257	chr6
622	*PTRF*	1.07E-261	0.81973884	0.494	0	3.15E-257	chr17
638	*RBM3*	2.17E-261	0.90801353	0.802	0.35	6.37E-257	chrX
321	*GLTSCR2*	4.26E-261	0.7588206	0.494	0.001	1.25E-256	chr19
843	*USMG5*	3.31E-260	0.66542969	0.493	0.001	9.71E-256	chr10
190	*COX6C*	1.01E-259	1.06899834	0.874	0.557	2.97E-255	chr8
439	*MFAP4*	4.39E-258	0.80728756	0.62	0.101	1.29E-253	chr17
806	*TNFRSF12A*	4.45E-258	1.06988968	0.565	0.052	1.30E-253	chr16
574	*PLOD1*	8.35E-256	0.72667909	0.682	0.16	2.45E-251	chr1
95	*C17orf89*	3.65E-255	0.73925819	0.485	0	1.07E-250	chr17
52	*ATP5F1*	6.15E-255	0.82539951	0.485	0.001	1.81E-250	chr1
222	*DCN*	2.21E-254	1.16206108	0.682	0.152	6.50E-250	chr12
84	*C11orf31*	1.26E-252	0.8289279	0.482	0.001	3.71E-248	chr11
815	*TRAM1*	2.52E-252	0.75098551	0.752	0.242	7.38E-248	chr8
299	*FGFR1*	6.06E-252	0.87494694	0.75	0.225	1.78E-247	chr8
693	*SELM*	5.26E-251	0.68158388	0.479	0	1.54E-246	chr22
78	*BNIP3*	6.40E-251	0.98916595	0.738	0.201	1.88E-246	chr10
836	*UFD1L*	3.88E-248	0.63434213	0.475	0.001	1.14E-243	chr22
473	*MXRA8*	8.35E-247	0.85653475	0.663	0.171	2.45E-242	chr1
738	*SNHG8*	8.49E-247	0.85542024	0.752	0.233	2.49E-242	chr4
342	*HLA-A*	2.09E-246	1.11112142	0.834	0.398	6.13E-242	chr6
699	*SEPW1*	7.07E-245	0.60526995	0.471	0.001	2.07E-240	chr19
751	*SPON2*	1.44E-243	0.89543698	0.48	0.009	4.21E-239	chr4
654	*RNH1*	2.11E-243	0.62342584	0.694	0.173	6.19E-239	chr11
49	*ATP5C1*	1.49E-242	0.61402305	0.466	0	4.36E-238	chr10
280	*FABP5*	4.12E-242	1.47073091	0.663	0.178	1.21E-237	chr8
284	*FAM195B*	7.67E-240	0.61166934	0.462	0	2.25E-235	chr17

75	*BGN*	3.11E-239	0.84924806	0.677	0.137	9.11E-235	chrX
36	*APOA1BP*	1.72E-238	0.53299114	0.46	0	5.06E-234	chr1
607	*PSAP*	4.71E-238	0.99836751	0.742	0.289	1.38E-233	chr10
766	*STRA13*	4.85E-238	0.6002639	0.46	0	1.42E-233	chr17
356	*HTRA1*	5.00E-237	0.85551869	0.735	0.216	1.47E-232	chr10
403	*LHFP*	1.25E-236	0.59630002	0.459	0.001	3.66E-232	chr13
553	*PGAM1*	1.36E-236	0.77863388	0.792	0.34	3.99E-232	chr10
695	*15-Sep*	2.38E-235	0.50507908	0.456	0	6.98E-231	chr1
113	*C7orf73*	4.13E-235	0.47483222	0.456	0.001	1.21E-230	chr7
787	*TMED9*	6.58E-235	0.72820563	0.732	0.243	1.93E-230	chr5
721	*SLC25A6*	6.57E-234	0.88186497	0.848	0.44	1.93E-229	chrX
562	*PHLDA3*	7.39E-234	0.56933144	0.554	0.064	2.17E-229	chr1
615	*PSMG3*	1.20E-233	0.54723477	0.645	0.137	3.51E-229	chr7
662	*RPS17*	2.53E-233	1.07552766	0.876	0.632	7.43E-229	chr15
334	*GUK1*	7.82E-232	0.79296907	0.816	0.364	2.30E-227	chr1
181	*COL6A2*	2.34E-230	1.52283938	0.77	0.31	6.87E-226	chr21
840	*UQCRH*	3.18E-230	0.79526055	0.829	0.445	9.34E-226	chr1
839	*UQCRFS1*	5.13E-230	0.72769596	0.669	0.181	1.51E-225	chr19
692	*SELK*	6.00E-228	0.46391356	0.446	0.001	1.76E-223	chr3
105	*C20orf24*	2.39E-227	0.48001645	0.445	0.001	7.02E-223	chr20
713	*SHMT2*	2.53E-227	0.52685666	0.625	0.118	7.43E-223	chr12
853	*VIMP*	4.21E-227	0.50772878	0.445	0.001	1.24E-222	chr15
858	*WBSCR22*	7.01E-227	0.4288672	0.445	0.001	2.06E-222	chr7
285	*FAM20C*	2.29E-226	0.49568964	0.541	0.057	6.72E-222	chr7
845	*UTP11L*	2.94E-226	0.47049888	0.442	0	8.62E-222	chr1
784	*TMED2*	5.75E-226	0.66866357	0.69	0.213	1.69E-221	chr12
437	*MESDC2*	1.54E-225	0.44388467	0.442	0.001	4.52E-221	chr15
616	*PTDSS1*	2.65E-224	0.50650772	0.586	0.098	7.78E-220	chr8
494	*NDUFA4L2*	5.07E-224	1.68873016	0.522	0.063	1.49E-219	chr12
770	*SYPL1*	2.91E-223	0.55367418	0.643	0.147	8.54E-219	chr7
536	*P4HB*	3.08E-223	0.84998202	0.819	0.443	9.04E-219	chr17
270	*ENO1*	3.16E-221	0.93666219	0.892	0.68	9.27E-217	chr1
479	*MYL9*	4.14E-221	0.75528127	0.54	0.059	1.21E-216	chr20
255	*EGLN2*	7.41E-221	0.46303749	0.474	0.021	2.17E-216	chr19
754	*SQSTM1*	9.45E-219	0.79747809	0.75	0.259	2.77E-214	chr5
313	*GBAS*	9.52E-219	0.40265115	0.432	0.001	2.79E-214	chr7
184	*COMMD6*	1.53E-218	0.66542993	0.779	0.279	4.50E-214	chr13
274	*EPB41L4A-AS1*	1.83E-218	0.54105399	0.65	0.141	5.37E-214	chr5
326	*GPAT2*	2.44E-218	0.46078397	0.432	0.001	7.15E-214	chr2

287	*FAM3C*	7.55E-218	0.53389597	0.636	0.137	2.22E-213	chr7
135	*CD44*	6.66E-216	0.54588626	0.635	0.129	1.95E-211	chr11
100	*C19orf60*	7.54E-215	0.39048194	0.426	0.001	2.21E-210	chr19
303	*FMOD*	2.71E-213	0.64149165	0.638	0.148	7.97E-209	chr1
329	*GRINA*	2.90E-213	0.58461304	0.668	0.178	8.51E-209	chr8
863	*WHSC1L1*	4.00E-213	0.3911322	0.423	0.001	1.17E-208	chr8
567	*PKM*	1.02E-212	0.84448194	0.843	0.554	3.01E-208	chr15
670	*RTFDC1*	2.54E-212	0.36205014	0.422	0.001	7.46E-208	chr20
214	*CYB5R3*	4.47E-211	0.59090251	0.656	0.185	1.31E-206	chr22
454	*MRPL37*	1.97E-209	0.47976605	0.572	0.108	5.79E-205	chr1
797	*TMEM261*	1.78E-207	0.3695065	0.413	0	5.23E-203	chr9
388	*KLF10*	2.22E-207	0.67783367	0.621	0.152	6.50E-203	chr8
875	*ZFAS1*	2.71E-207	0.92724303	0.858	0.436	7.96E-203	chr20
197	*CPSF3L*	1.25E-206	0.35256451	0.412	0	3.68E-202	chr1
10	*ADSL*	3.71E-206	0.61837374	0.496	0.056	1.09E-201	chr22
107	*C4orf3*	4.47E-206	0.55035151	0.71	0.209	1.31E-201	chr4
420	*LY6E*	1.27E-205	1.06104647	0.797	0.375	3.72E-201	chr8
298	*FDX1*	2.03E-204	0.46155668	0.502	0.057	5.96E-200	chr11
290	*FAM92A1*	2.63E-204	0.44623346	0.408	0	7.73E-200	chr8
461	*MRPS6*	2.99E-204	0.8688456	0.752	0.278	8.77E-200	chr21
792	*TMEM173*	7.53E-204	0.53514873	0.556	0.095	2.21E-199	chr5
719	*SLC16A3*	6.81E-203	0.61895549	0.558	0.094	2.00E-198	chr17
640	*RCN1*	1.47E-202	0.74817909	0.679	0.222	4.31E-198	chr11
153	*CECR5*	3.72E-201	0.35271437	0.403	0	1.09E-196	chr22
103	*C1QTNF3*	4.91E-201	0.63345583	0.528	0.075	1.44E-196	chr5
643	*RGS3*	7.99E-201	0.59363077	0.637	0.163	2.34E-196	chr9
88	*C14orf1*	1.58E-200	0.37301699	0.402	0	4.64E-196	chr14
563	*PHPT1*	3.43E-200	0.6473332	0.759	0.311	1.01E-195	chr9
684	*SDC2*	4.33E-200	0.72075902	0.718	0.275	1.27E-195	chr8
327	*GPNMB*	3.33E-199	0.75364037	0.436	0.018	9.77E-195	chr7
258	*EI24*	1.65E-197	0.49016165	0.629	0.157	4.85E-193	chr11
683	*SCPEP1*	2.07E-197	0.43481408	0.545	0.091	6.08E-193	chr17
210	*CTSL*	3.18E-197	0.50382456	0.538	0.085	9.33E-193	chr9
92	*C16orf13*	3.41E-197	0.33372098	0.397	0	1.00E-192	chr16
408	*LINC00657*	3.41E-197	0.33153085	0.397	0	1.00E-192	chr20
522	*NUDT14*	3.12E-196	0.50973343	0.553	0.107	9.16E-192	chr14
406	*LINC00493*	4.27E-196	0.32484625	0.397	0.001	1.25E-191	chr20
309	*FXYD6*	1.07E-195	0.4775172	0.553	0.102	3.15E-191	chr11
826	*TUBB6*	3.28E-195	0.51423635	0.566	0.113	9.63E-191	chr18
85	*C11orf73*	9.14E-195	0.32659491	0.394	0.001	2.68E-190	chr11

62	*ATP5SL*	1.04E-194	0.313675	0.393	0	3.07E-190	chr19
384	*KDELR2*	1.50E-194	0.70225523	0.813	0.418	4.39E-190	chr7
418	*LTBR*	1.74E-194	0.3624099	0.401	0.004	5.12E-190	chr12
186	*COPRS*	3.20E-194	0.61574141	0.562	0.119	9.38E-190	chr17
201	*CRISPLD1*	9.62E-194	0.68224347	0.626	0.156	2.82E-189	chr8
413	*LOXL1*	1.90E-193	0.49814326	0.585	0.118	5.57E-189	chr15
224	*DDIT4*	1.51E-192	1.58347951	0.704	0.294	4.42E-188	chr10
801	*TMEM55A*	2.25E-192	0.48109185	0.391	0.001	6.60E-188	chr8
244	*DUSP2*	5.97E-192	0.80077351	0.464	0.047	1.75E-187	chr2
225	*DDT*	6.74E-192	0.70391519	0.691	0.245	1.98E-187	chr22
685	*SDCBP*	6.02E-191	0.75007265	0.766	0.372	1.77E-186	chr8
822	*TSPAN4*	1.50E-190	0.63901847	0.584	0.136	4.41E-186	chr11
208	*CTSD*	1.58E-190	0.59992396	0.673	0.209	4.64E-186	chr11
187	*COPZ1*	6.32E-190	0.44629682	0.623	0.157	1.85E-185	chr12
561	*PHLDA1*	1.64E-189	0.51964439	0.492	0.059	4.82E-185	chr12
20	*ALDOA*	3.24E-189	0.93411885	0.642	0.209	9.52E-185	chr16
64	*ATP6V0E1*	4.18E-189	0.57219093	0.723	0.263	1.23E-184	chr5
353	*HSPB1*	4.48E-189	0.97199774	0.835	0.517	1.31E-184	chr7
102	*C1orf43*	6.50E-189	0.5074949	0.659	0.201	1.91E-184	chr1
235	*DKK3*	7.04E-189	0.46081228	0.431	0.026	2.07E-184	chr11
838	*UQCR11*	1.63E-188	0.6618102	0.796	0.402	4.79E-184	chr19
386	*KIAA0101*	8.37E-188	0.62043731	0.383	0.001	2.46E-183	chr15
156	*CHCHD2*	1.82E-187	0.61200529	0.896	0.68	5.33E-183	chr7
207	*CTSB*	1.91E-187	0.66294768	0.647	0.193	5.60E-183	chr8
648	*RHOC*	2.81E-187	0.60039312	0.711	0.26	8.24E-183	chr1
596	*PPP2R4*	2.45E-186	0.29172369	0.381	0.001	7.18E-182	chr9
700	*SERPINE2*	1.33E-185	0.90747631	0.638	0.178	3.89E-181	chr2
841	*UQCRQ*	2.00E-185	0.63539226	0.787	0.387	5.87E-181	chr5
70	*AXL*	2.14E-185	0.43170716	0.49	0.063	6.27E-181	chr19
873	*ZFAND1*	3.05E-185	0.36009825	0.524	0.086	8.93E-181	chr8
179	*COL5A1*	3.05E-185	0.50688523	0.626	0.15	8.94E-181	chr9
155	*CFH*	8.98E-185	0.36042667	0.396	0.009	2.63E-180	chr1
645	*RHBDD2*	6.95E-184	0.40503892	0.541	0.103	2.04E-179	chr7
421	*LY6K*	1.63E-183	0.4325352	0.378	0.002	4.77E-179	chr8
491	*NDUFA11*	3.65E-183	0.63021497	0.762	0.353	1.07E-178	chr19
282	*FAM103A1*	7.26E-183	0.27876336	0.374	0	2.13E-178	chr15
595	*PPP2R1A*	2.97E-182	0.49937239	0.7	0.245	8.71E-178	chr19
532	*OXA1L*	3.34E-182	0.41037816	0.595	0.139	9.79E-178	chr14
409	*LITAF*	3.91E-182	0.56118718	0.718	0.248	1.15E-177	chr16
94	*C17orf62*	4.65E-182	0.30814401	0.372	0	1.36E-177	chr17

477	*MYL12A*	7.22E-182	0.6320566	0.693	0.274	2.12E-177	chr18
831	*TXNDC12*	8.49E-182	0.46384279	0.603	0.153	2.49E-177	chr1
397	*LAPTM4A*	8.88E-182	0.72723819	0.813	0.491	2.61E-177	chr2
735	*SNAI2*	2.05E-181	0.60929395	0.53	0.107	6.02E-177	chr8
83	*BSG*	9.05E-181	0.72305135	0.756	0.352	2.66E-176	chr19
281	*FAM101B*	1.19E-180	0.35840491	0.37	0	3.48E-176	chr17
604	*PRR13*	7.75E-180	0.42330211	0.58	0.137	2.27E-175	chr12
283	*FAM127A*	1.20E-179	0.29318209	0.368	0	3.51E-175	chrX
525	*OAF*	1.86E-179	0.53766306	0.61	0.151	5.47E-175	chr11
601	*PRKCDBP*	1.90E-179	0.45893556	0.368	0	5.56E-175	chr11
415	*LSM1*	2.18E-179	0.38801266	0.572	0.128	6.39E-175	chr8
169	*CLEC2B*	2.36E-179	0.51429179	0.419	0.024	6.93E-175	chr12
689	*SDHD*	3.37E-179	0.31548143	0.418	0.025	9.89E-175	chr11
807	*TNFRSF1A*	3.98E-179	0.42454654	0.525	0.093	1.17E-174	chr12
463	*MSC*	4.41E-179	0.61905904	0.386	0.011	1.29E-174	chr8
233	*DGCR6L*	4.64E-179	0.41272021	0.539	0.107	1.36E-174	chr22
369	*IL11RA*	5.59E-179	0.49319392	0.517	0.084	1.64E-174	chr9
657	*RP11-386G11.10*	5.59E-179	0.40532095	0.369	0.001	1.64E-174	chr12
484	*NAB2*	9.87E-179	0.35408277	0.446	0.042	2.89E-174	chr12
65	*ATP6V1E1*	1.04E-178	0.43680928	0.568	0.125	3.06E-174	chr22
501	*NDUFC2*	1.84E-178	0.63444414	0.746	0.334	5.41E-174	chr11
696	*SEPN1*	4.76E-178	0.27942505	0.366	0	1.40E-173	chr1
555	*PGF*	9.77E-178	0.76096175	0.656	0.23	2.87E-173	chr14
422	*LYPLA1*	1.02E-177	0.45964743	0.593	0.151	3.00E-173	chr8
302	*FKBP10*	1.42E-177	0.56168569	0.647	0.197	4.16E-173	chr17
16	*AK4*	2.81E-177	0.34313267	0.434	0.036	8.24E-173	chr1
796	*TMEM230*	3.04E-177	0.42810754	0.643	0.183	8.92E-173	chr20
549	*PEF1*	4.17E-177	0.39997056	0.574	0.136	1.22E-172	chr1
424	*MAF1*	9.85E-177	0.48618182	0.686	0.232	2.89E-172	chr8
679	*SCARB2*	3.09E-176	0.37452038	0.5	0.075	9.05E-172	chr4
448	*MMP24-AS1*	3.38E-176	0.30203967	0.364	0.001	9.92E-172	chr20
508	*NNAT*	4.85E-176	2.63589669	0.397	0.026	1.42E-171	chr20
264	*ELN*	5.08E-176	0.66112461	0.551	0.116	1.49E-171	chr7
788	*TMEM109*	1.30E-175	0.41507345	0.568	0.133	3.81E-171	chr11
653	*RNF212*	2.87E-175	0.34836221	0.382	0.009	8.42E-171	chr4
173	*CNIH1*	3.55E-175	0.39575641	0.619	0.159	1.04E-170	chr14
757	*SSNA1*	6.72E-175	0.47501144	0.639	0.196	1.97E-170	chr9
531	*OSTC*	1.08E-174	0.57549971	0.759	0.317	3.18E-170	chr4
385	*KDELR3*	1.19E-174	0.41206814	0.493	0.079	3.50E-170	chr22

857	*WBSCR16*	1.83E-174	0.25340275	0.361	0.001	5.37E-170	chr7
372	*IMPAD1*	1.93E-174	0.47491121	0.571	0.14	5.67E-170	chr8
110	*C7orf49*	4.52E-174	0.27652564	0.359	0	1.32E-169	chr7
802	*TMEM70*	8.82E-173	0.46401582	0.591	0.155	2.59E-168	chr8
13	*AGPAT2*	2.32E-172	0.40271109	0.603	0.156	6.79E-168	chr9
228	*DDX54*	1.63E-171	0.32699702	0.508	0.086	4.79E-167	chr12
658	*RP11-395G23.3*	1.67E-171	0.33956679	0.355	0	4.89E-167	chr8
544	*PDLIM3*	7.08E-171	0.56236994	0.383	0.017	2.08E-166	chr4
15	*AK1*	1.79E-170	0.40853922	0.494	0.084	5.26E-166	chr9
9	*ADPRHL2*	3.15E-170	0.33729624	0.514	0.091	9.23E-166	chr1
192	*COX7A2L*	3.74E-170	0.57097475	0.744	0.337	1.10E-165	chr2
694	*SELT*	7.27E-170	0.25456323	0.353	0.001	2.13E-165	chr3
756	*SRSF9*	8.11E-170	0.59605916	0.763	0.372	2.38E-165	chr12
147	*CDK4*	9.04E-170	0.52931938	0.686	0.26	2.65E-165	chr12
157	*CHID1*	9.76E-170	0.38844424	0.573	0.135	2.86E-165	chr11
540	*PARVB*	1.11E-169	0.33059496	0.46	0.059	3.27E-165	chr22
130	*CCND3*	1.38E-169	0.39570998	0.514	0.097	4.06E-165	chr6
139	*CD82*	6.61E-169	0.33409535	0.392	0.022	1.94E-164	chr11
748	*SPG21*	1.30E-168	0.36790145	0.542	0.113	3.82E-164	chr15
140	*CD99*	2.36E-168	0.75489411	0.753	0.31	6.92E-164	chrX
288	*FAM46A*	2.66E-168	0.34161612	0.351	0.001	7.79E-164	chr6
3	*ABCF2*	5.83E-168	0.27897416	0.371	0.01	1.71E-163	chr7
304	*FNTA*	1.33E-167	0.35665533	0.561	0.127	3.89E-163	chr8
675	*S100A2*	1.46E-166	0.73238921	0.364	0.009	4.29E-162	chr1
432	*MDH2*	2.45E-166	0.5273288	0.736	0.313	7.20E-162	chr7
872	*ZDHHC4*	6.31E-166	0.36684386	0.538	0.113	1.85E-161	chr7
149	*CDKN1A*	7.02E-166	0.49984332	0.536	0.114	2.06E-161	chr6
504	*NEK6*	1.39E-165	0.32402013	0.466	0.067	4.09E-161	chr9
151	*CDKN2C*	1.85E-165	0.43758845	0.494	0.09	5.43E-161	chr1
185	*COMT*	3.95E-165	0.2922044	0.462	0.063	1.16E-160	chr22
82	*BRK1*	4.84E-164	0.82556526	0.731	0.356	1.42E-159	chr3
605	*PRR5*	5.17E-164	0.35712212	0.449	0.06	1.52E-159	chr22
528	*OLFML3*	6.57E-164	0.44737404	0.514	0.101	1.93E-159	chr1
762	*ST3GAL4*	8.89E-164	0.31467404	0.394	0.027	2.61E-159	chr11
279	*FABP3*	1.58E-163	0.44074999	0.369	0.013	4.63E-159	chr1
830	*TXN2*	1.60E-163	0.43328547	0.654	0.212	4.70E-159	chr22
46	*ASPH*	2.56E-163	0.46193544	0.621	0.176	7.51E-159	chr8
8	*ADM*	3.01E-163	0.68717324	0.556	0.133	8.84E-159	chr11
712	*SHISA5*	4.30E-163	0.42832808	0.594	0.165	1.26E-158	chr3
781	*TMBIM6*	8.56E-163	0.56506751	0.776	0.418	2.51E-158	chr12

603	*PRNP*	2.87E-162	0.39696952	0.465	0.071	8.41E-158	chr20
152	*CEBPD*	3.05E-162	1.06027707	0.648	0.252	8.94E-158	chr8
747	*SPG20*	3.70E-162	0.25405838	0.34	0.001	1.09E-157	chr13
829	*TXN*	8.03E-162	0.58501208	0.776	0.401	2.36E-157	chr9
798	*TMEM43*	8.09E-162	0.31761449	0.445	0.057	2.38E-157	chr3
780	*TM2D1*	8.31E-162	0.3531989	0.576	0.138	2.44E-157	chr1
275	*EPHX1*	9.26E-162	0.39114301	0.448	0.06	2.72E-157	chr1
587	*POP4*	1.34E-161	0.26000377	0.411	0.037	3.94E-157	chr19
30	*ANXA5*	3.91E-161	0.67529011	0.734	0.297	1.15E-156	chr4
868	*YARS*	4.24E-161	0.38140493	0.51	0.1	1.25E-156	chr1
624	*PVRL2*	5.47E-161	0.26184762	0.337	0	1.61E-156	chr19
768	*SUN1*	6.19E-161	0.53603043	0.507	0.095	1.82E-156	chr7
818	*TRIOBP*	9.01E-161	0.34116653	0.484	0.083	2.64E-156	chr22
419	*LUM*	9.33E-161	1.11413273	0.436	0.062	2.74E-156	chr12
717	*SIX1*	1.16E-160	0.73355834	0.623	0.22	3.40E-156	chr14
803	*TMEM9*	1.92E-160	0.44962876	0.574	0.156	5.62E-156	chr1
449	*MOXD1*	3.31E-160	0.37060589	0.429	0.052	9.72E-156	chr6
315	*GGH*	5.00E-160	0.69694807	0.566	0.175	1.47E-155	chr8
115	*C9orf142*	1.89E-159	0.26582085	0.334	0	5.54E-155	chr9
564	*PIH1D1*	4.04E-159	0.29358501	0.426	0.045	1.19E-154	chr19
621	*PTPMT1*	6.24E-159	0.37568489	0.506	0.105	1.83E-154	chr11
305	*FRZB*	8.20E-159	0.45365551	0.352	0.009	2.41E-154	chr2
816	*TRIAP1*	1.08E-158	0.33121157	0.507	0.099	3.16E-154	chr12
697	*SEPP1*	1.10E-158	0.65140948	0.333	0	3.24E-154	chr5
859	*WDR13*	1.25E-158	0.31214642	0.477	0.079	3.66E-154	chrX
40	*ARL5A*	1.35E-158	0.34058619	0.528	0.116	3.96E-154	chr2
174	*CNN3*	2.03E-158	0.70198483	0.74	0.355	5.96E-154	chr1
4	*ACADVL*	4.25E-158	0.43509164	0.574	0.144	1.25E-153	chr17
443	*MINOS1*	1.14E-157	0.66443365	0.618	0.24	3.34E-153	chr1
194	*CPE*	1.90E-157	0.72651591	0.69	0.261	5.58E-153	chr4
455	*MRPL41*	5.54E-157	0.40619762	0.607	0.189	1.63E-152	chr9
440	*MFGE8*	6.36E-157	0.4377188	0.553	0.137	1.87E-152	chr15
680	*SCARF2*	1.01E-156	0.32944038	0.402	0.038	2.95E-152	chr22
219	*DARS*	1.42E-156	0.36643336	0.595	0.157	4.15E-152	chr2
552	*PEX2*	1.63E-156	0.35038597	0.574	0.144	4.79E-152	chr8
109	*C6orf48*	1.82E-156	0.74886502	0.674	0.305	5.35E-152	chr6
112	*C7orf55*	3.10E-156	0.27637381	0.33	0.001	9.11E-152	chr7
213	*CYB5R1*	3.23E-156	0.38048344	0.417	0.048	9.48E-152	chr1
785	*TMED3*	3.79E-156	0.38913049	0.648	0.205	1.11E-151	chr15
705	*SERTAD3*	6.33E-156	0.30534648	0.46	0.072	1.86E-151	chr19

759	*SSR2*	6.60E-156	0.64741441	0.815	0.43	1.94E-151	chr1
647	*RHOBTB3*	9.93E-156	0.54083269	0.657	0.248	2.91E-151	chr5
771	*TAF1D*	1.04E-155	0.4543275	0.637	0.195	3.04E-151	chr11
867	*XRCC6*	1.18E-155	0.60659257	0.735	0.32	3.46E-151	chr22
876	*ZFYVE21*	2.11E-155	0.29214716	0.486	0.087	6.20E-151	chr14
101	*C19orf70*	2.30E-155	0.40318035	0.582	0.167	6.74E-151	chr19
23	*ANAPC16*	2.74E-155	0.44437519	0.653	0.224	8.03E-151	chr10
394	*LAMP1*	3.47E-155	0.46866837	0.662	0.234	1.02E-150	chr13
509	*NNMT*	3.99E-155	0.58480447	0.372	0.022	1.17E-150	chr11
183	*COMMD5*	6.04E-155	0.32328746	0.5	0.096	1.77E-150	chr8
652	*RNF187*	7.34E-155	0.42211331	0.63	0.191	2.15E-150	chr1
594	*PPP1R15A*	8.90E-155	0.73260616	0.675	0.26	2.61E-150	chr19
148	*CDK6*	9.71E-154	0.37902021	0.53	0.122	2.85E-149	chr7
426	*MAGED2*	2.09E-153	0.95374726	0.735	0.375	6.13E-149	chrX
25	*ANGPTL4*	7.75E-153	0.76648693	0.523	0.119	2.27E-148	chr19
296	*FBXW5*	1.34E-152	0.33190269	0.543	0.133	3.93E-148	chr9
215	*CYBRD1*	1.43E-152	0.28151924	0.391	0.036	4.19E-148	chr2
545	*PDLIM4*	1.57E-152	0.47054457	0.627	0.202	4.59E-148	chr5
745	*SPATC1L*	3.65E-152	0.32722322	0.442	0.066	1.07E-147	chr21
79	*BOC*	3.82E-152	0.45486888	0.537	0.132	1.12E-147	chr3
150	*CDKN2A*	3.88E-152	0.65235759	0.325	0.002	1.14E-147	chr9
444	*MKKS*	7.01E-152	0.27993622	0.452	0.071	2.06E-147	chr20
535	*P4HA2*	1.13E-151	0.37684844	0.557	0.144	3.33E-147	chr5
614	*PSMG2*	1.24E-151	0.32445638	0.527	0.118	3.65E-147	chr18
163	*CISD2*	1.39E-151	0.30431345	0.527	0.118	4.07E-147	chr4
460	*MRPS28*	1.55E-151	0.28120423	0.448	0.067	4.55E-147	chr8
618	*PTGES2*	3.57E-151	0.31212296	0.472	0.089	1.05E-146	chr9
257	*EHD2*	4.73E-151	0.32728586	0.435	0.06	1.39E-146	chr19
805	*TMX2*	8.03E-151	0.3086041	0.479	0.093	2.36E-146	chr11
263	*EIF5A*	8.05E-151	0.61862458	0.733	0.388	2.36E-146	chr17
231	*DES*	1.03E-150	0.91698014	0.321	0.001	3.03E-146	chr2
182	*COL6A3*	1.53E-150	0.70763877	0.585	0.164	4.50E-146	chr2
430	*MCOLN3*	3.14E-150	0.39719486	0.393	0.041	9.22E-146	chr1
204	*CSTB*	3.57E-150	0.48688221	0.724	0.278	1.05E-145	chr21
355	*HSPG2*	4.32E-150	0.45811381	0.49	0.099	1.27E-145	chr1
2	*A2M*	5.12E-150	0.45981298	0.366	0.022	1.50E-145	chr12
29	*ANXA2*	5.57E-150	0.85719364	0.816	0.495	1.63E-145	chr15
626	*PXMP2*	5.63E-150	0.32367117	0.477	0.089	1.65E-145	chr12
354	*HSPB6*	5.83E-150	0.25448382	0.323	0.002	1.71E-145	chr19
189	*COX6A1*	8.91E-150	0.53091851	0.8	0.523	2.61E-145	chr12

617	*PTGDS*	1.16E-149	0.90626002	0.33	0.007	3.41E-145	chr9
773	*TATDN1*	2.33E-149	0.39792519	0.652	0.23	6.83E-145	chr8
469	*MT1X*	3.76E-149	0.81305954	0.691	0.267	1.10E-144	chr16
392	*KXD1*	4.84E-149	0.32666606	0.57	0.151	1.42E-144	chr19
230	*DEGS1*	1.13E-148	0.35870189	0.459	0.08	3.30E-144	chr1
810	*TOMM22*	1.25E-148	0.44920423	0.606	0.199	3.67E-144	chr22
250	*ECHS1*	1.47E-148	0.5494558	0.658	0.254	4.31E-144	chr10
882	*ZYX*	1.69E-148	0.4236489	0.57	0.169	4.97E-144	chr7
848	*VAMP3*	1.88E-148	0.25702943	0.417	0.052	5.52E-144	chr1
438	*MFAP2*	2.09E-148	0.55773505	0.746	0.328	6.12E-144	chr1
452	*MRPL13*	2.13E-148	0.44241323	0.664	0.25	6.26E-144	chr8
108	*C5orf15*	2.47E-148	0.27211327	0.398	0.043	7.24E-144	chr5
790	*TMEM123*	3.00E-148	0.33677557	0.422	0.058	8.81E-144	chr11
436	*MED30*	4.79E-148	0.38954932	0.512	0.12	1.41E-143	chr8
325	*GPAA1*	5.37E-148	0.42437447	0.672	0.234	1.57E-143	chr8
842	*URM1*	6.36E-148	0.28038316	0.503	0.107	1.87E-143	chr9
247	*EBAG9*	9.19E-148	0.30161485	0.531	0.122	2.70E-143	chr8
512	*NPM3*	1.83E-147	0.35381069	0.545	0.144	5.36E-143	chr10
524	*NXPH4*	1.93E-147	0.41927836	0.375	0.033	5.66E-143	chr12
472	*MTHFD2*	2.28E-147	0.43631277	0.539	0.139	6.69E-143	chr2
97	*C19orf12*	5.95E-147	0.25211965	0.372	0.029	1.75E-142	chr19
741	*SNRPN*	6.77E-147	0.60035808	0.592	0.207	1.98E-142	chr15
598	*PRAF2*	7.68E-147	0.30275983	0.462	0.084	2.25E-142	chrX
795	*TMEM222*	9.79E-147	0.29368742	0.477	0.092	2.87E-142	chr1
599	*PRAME*	2.50E-146	0.59638411	0.576	0.188	7.32E-142	chr22
660	*RPA3*	3.93E-146	0.3883885	0.568	0.16	1.15E-141	chr7
374	*ISLR*	7.19E-146	0.43770006	0.581	0.165	2.11E-141	chr15
162	*CISD1*	8.59E-146	0.32451863	0.484	0.099	2.52E-141	chr10
828	*TUG1*	1.25E-145	0.29008292	0.337	0.011	3.68E-141	chr22
569	*PLAGL1*	1.34E-145	0.33714539	0.469	0.09	3.92E-141	chr6
456	*MRPL55*	1.48E-145	0.36350998	0.606	0.184	4.33E-141	chr1
655	*RNMT*	4.82E-145	0.51720943	0.607	0.183	1.41E-140	chr18
736	*SNAPIN*	7.88E-145	0.30765684	0.484	0.098	2.31E-140	chr1
28	*ANXA11*	1.07E-144	0.32874237	0.539	0.14	3.15E-140	chr10
586	*PON2*	2.64E-144	0.26980173	0.424	0.061	7.76E-140	chr7
764	*STOM*	4.05E-144	0.26970604	0.427	0.062	1.19E-139	chr9
649	*RNASEH2A*	9.69E-144	0.27155968	0.373	0.034	2.84E-139	chr19
41	*ARL6IP4*	1.36E-143	0.57177384	0.768	0.463	3.99E-139	chr12
470	*MTFP1*	2.33E-143	0.27527746	0.331	0.011	6.83E-139	chr22
732	*SMIM3*	3.72E-143	0.35482817	0.389	0.044	1.09E-138	chr5

855	*VOPP1*	4.08E-143	0.34064707	0.549	0.141	1.20E-138	chr7
427	*MAN1B1*	1.39E-142	0.28171186	0.434	0.067	4.08E-138	chr9
74	*BCAP31*	1.52E-142	0.38676314	0.653	0.222	4.46E-138	chrX
165	*CITED4*	1.76E-142	0.42723461	0.439	0.075	5.16E-138	chr1
330	*GRN*	2.55E-142	0.60628832	0.649	0.248	7.49E-138	chr17
557	*PGM1*	5.14E-142	0.3035614	0.507	0.116	1.51E-137	chr1
170	*CLNS1A*	6.83E-142	0.38454022	0.633	0.22	2.01E-137	chr11
416	*LSM10*	3.44E-141	0.31394037	0.537	0.143	1.01E-136	chr1
733	*SMIM7*	5.23E-141	0.38031054	0.614	0.194	1.53E-136	chr19
492	*NDUFA13*	5.54E-141	0.54603226	0.769	0.417	1.63E-136	chr19
24	*ANGPTL2*	7.80E-141	0.35992391	0.455	0.083	2.29E-136	chr9
678	*SAT2*	9.89E-141	0.45217208	0.682	0.252	2.90E-136	chr17
442	*MGP*	1.02E-140	1.21926532	0.789	0.361	2.99E-136	chr12
630	*RAB34*	1.07E-140	0.4592966	0.735	0.325	3.15E-136	chr17
363	*IFI6*	1.26E-140	0.40250312	0.409	0.06	3.70E-136	chr1
480	*MYLPF*	2.70E-140	1.22964522	0.329	0.016	7.93E-136	chr16
154	*CERCAM*	3.60E-140	0.26125473	0.396	0.05	1.06E-135	chr9
239	*DPM2*	4.21E-140	0.27706269	0.497	0.109	1.24E-135	chr9
578	*PLXNB2*	8.11E-140	0.35211788	0.488	0.105	2.38E-135	chr22
126	*CCDC8*	1.19E-139	0.26826914	0.445	0.078	3.50E-135	chr19
488	*NDN*	1.89E-139	0.56636558	0.654	0.266	5.53E-135	chr15
119	*CAPN2*	3.67E-139	0.318975	0.515	0.123	1.08E-134	chr1
767	*SULF1*	1.03E-138	0.34704569	0.376	0.039	3.03E-134	chr8
534	*P4HA1*	1.41E-138	0.39828637	0.626	0.196	4.14E-134	chr10
743	*SPAG7*	1.50E-138	0.28309254	0.599	0.181	4.39E-134	chr17
362	*IFI16*	2.71E-138	0.45482256	0.43	0.074	7.96E-134	chr1
393	*LACTB2*	3.35E-138	0.3359975	0.441	0.081	9.82E-134	chr8
682	*SCOC*	7.61E-138	0.26339295	0.538	0.14	2.23E-133	chr4
704	*SERTAD1*	1.88E-137	0.42594768	0.595	0.188	5.53E-133	chr19
121	*CAV1*	1.95E-137	0.40530892	0.478	0.093	5.73E-133	chr7
405	*LINC00152*	2.35E-137	0.37465756	0.295	0	6.90E-133	chr2
851	*VDAC1*	2.53E-137	0.48092979	0.709	0.316	7.42E-133	chr5
118	*CAMLG*	2.64E-137	0.35310932	0.617	0.194	7.76E-133	chr5
677	*SAMM50*	4.53E-137	0.32908873	0.531	0.145	1.33E-132	chr22
159	*CHRNA1*	8.23E-137	0.62827066	0.316	0.012	2.42E-132	chr2
529	*OS9*	2.76E-136	0.38521044	0.631	0.218	8.10E-132	chr12
99	*C19orf53*	2.86E-136	0.43358277	0.763	0.356	8.40E-132	chr19
339	*HILPDA*	3.23E-136	0.4578298	0.532	0.138	9.49E-132	chr7
708	*SH3GL1*	5.26E-136	0.33086085	0.542	0.148	1.54E-131	chr19
212	*CXXC5*	6.28E-136	0.39613524	0.584	0.18	1.84E-131	chr5

686	*SDF2L1*	1.12E-135	0.44331254	0.579	0.194	3.29E-131	chr22
217	*CYSTM1*	1.13E-135	0.31843308	0.423	0.071	3.30E-131	chr5
627	*PYURF*	1.19E-135	0.31031187	0.576	0.168	3.50E-131	chr4
663	*RPS19BP1*	1.31E-135	0.43066613	0.707	0.303	3.83E-131	chr22
203	*CRTAP*	1.35E-135	0.3571216	0.591	0.183	3.97E-131	chr3
814	*TPM2*	3.40E-135	0.58307698	0.771	0.417	9.98E-131	chr9
457	*MRPS12*	3.49E-135	0.30214162	0.57	0.169	1.02E-130	chr19
450	*MPG*	4.18E-135	0.32561268	0.551	0.157	1.23E-130	chr16
502	*NDUFS5*	4.96E-135	0.62061342	0.774	0.43	1.46E-130	chr1
336	*HEXA*	7.52E-135	0.30995123	0.538	0.145	2.21E-130	chr15
778	*THY1*	1.30E-134	0.518432	0.475	0.118	3.83E-130	chr11
114	*C8orf33*	1.91E-134	0.38861495	0.68	0.239	5.61E-130	chr8
687	*SDHAF2*	3.11E-134	0.29950341	0.477	0.106	9.12E-130	chr11
481	*MYOD1*	4.93E-134	0.55675501	0.296	0.003	1.45E-129	chr11
370	*IL32*	5.13E-134	0.48741346	0.315	0.013	1.51E-129	chr16
709	*SHC1*	5.32E-134	0.35575513	0.561	0.168	1.56E-129	chr1
441	*MGMT*	5.71E-134	0.30359788	0.555	0.155	1.68E-129	chr10
295	*FBXO7*	7.57E-134	0.33037822	0.523	0.14	2.22E-129	chr22
471	*MTFR1L*	1.57E-133	0.30082807	0.503	0.127	4.61E-129	chr1
597	*PPT1*	2.04E-133	0.38963394	0.593	0.203	5.99E-129	chr1
793	*TMEM176B*	2.13E-133	0.41928829	0.373	0.047	6.26E-129	chr7
548	*PEBP1*	2.19E-133	0.55443232	0.744	0.359	6.44E-129	chr12
706	*SGCA*	2.80E-133	0.61426174	0.29	0.001	8.20E-129	chr17
750	*SPOCK2*	7.81E-133	0.73148223	0.32	0.018	2.29E-128	chr10
134	*CD320*	7.83E-133	0.3527842	0.573	0.176	2.30E-128	chr19
879	*ZNF511*	9.32E-133	0.31232751	0.505	0.129	2.73E-128	chr10
667	*RRS1*	1.78E-132	0.25637243	0.455	0.091	5.22E-128	chr8
345	*HLA-E*	4.16E-132	0.54144809	0.657	0.252	1.22E-127	chr6
740	*SNRNP40*	4.53E-132	0.25552191	0.514	0.133	1.33E-127	chr1
755	*SRM*	4.79E-132	0.51434452	0.704	0.317	1.40E-127	chr1
106	*C22orf39*	5.88E-132	0.25892496	0.429	0.076	1.73E-127	chr22
338	*HIGD2A*	2.62E-131	0.3456358	0.627	0.215	7.68E-127	chr5
702	*SERPINH1*	3.63E-131	0.61009802	0.777	0.39	1.07E-126	chr11
556	*PGK1*	5.49E-131	0.53941441	0.804	0.462	1.61E-126	chrX
131	*CCNI*	7.15E-131	0.52354931	0.804	0.475	2.10E-126	chr4
291	*FASTK*	1.03E-130	0.32383058	0.572	0.179	3.03E-126	chr7
482	*MZT2A*	1.18E-130	0.42727375	0.749	0.336	3.46E-126	chr2
410	*LMCD1*	1.53E-130	0.31160448	0.419	0.074	4.49E-126	chr3
133	*CD164*	1.60E-130	0.39019627	0.677	0.274	4.69E-126	chr6
350	*HNRNPA1*	2.05E-130	0.54121878	0.866	0.645	6.00E-126	chr12

371	*IMPA2*	2.21E-130	0.36114514	0.405	0.07	6.48E-126	chr18
497	*NDUFB2*	2.40E-130	0.51419285	0.765	0.394	7.04E-126	chr7
789	*TMEM119*	4.97E-130	0.2523167	0.331	0.026	1.46E-125	chr12
846	*UTP23*	5.57E-130	0.32496031	0.563	0.163	1.63E-125	chr8
226	*DDX18*	7.55E-130	0.38232414	0.705	0.262	2.21E-125	chr2
716	*SIVA1*	1.31E-129	0.46135681	0.742	0.341	3.84E-125	chr14
265	*ELP5*	1.60E-129	0.28167948	0.423	0.077	4.70E-125	chr17
429	*MARVELD1*	1.74E-129	0.3134841	0.512	0.131	5.11E-125	chr10
777	*TGIF1*	2.29E-129	0.50728308	0.48	0.13	6.72E-125	chr18
301	*FHL2*	2.55E-129	0.48761389	0.413	0.078	7.49E-125	chr2
794	*TMEM203*	5.50E-129	0.25666202	0.545	0.15	1.61E-124	chr9
18	*AKR1C3*	5.86E-129	0.30403611	0.38	0.054	1.72E-124	chr10
266	*EMC10*	1.20E-128	0.33815281	0.619	0.209	3.51E-124	chr19
715	*SIRT2*	3.24E-128	0.28722035	0.464	0.106	9.51E-124	chr19
241	*DRG1*	5.07E-128	0.37684024	0.507	0.144	1.49E-123	chr22
12	*AFG3L2*	5.39E-128	0.28294785	0.456	0.1	1.58E-123	chr18
1	*A1BG*	6.87E-128	0.27927648	0.536	0.145	2.02E-123	chr19
381	*KAZALD1*	1.29E-127	0.28518294	0.429	0.083	3.78E-123	chr10
668	*RSL24D1*	2.81E-127	0.33606067	0.659	0.236	8.25E-123	chr15
42	*ARL6IP5*	3.17E-127	0.34540807	0.589	0.19	9.30E-123	chr3
659	*RPA2*	5.69E-127	0.29004085	0.519	0.141	1.67E-122	chr1
377	*ITPA*	9.34E-127	0.26568901	0.561	0.168	2.74E-122	chr20
566	*PKIG*	1.00E-126	0.25987392	0.498	0.124	2.94E-122	chr20
486	*NBL1*	1.53E-126	0.41586975	0.512	0.146	4.50E-122	chr1
499	*NDUFB5*	3.14E-126	0.30172387	0.58	0.187	9.21E-122	chr3
158	*CHPF*	4.14E-126	0.29779571	0.504	0.132	1.21E-121	chr2
861	*WDR45*	4.37E-126	0.26799416	0.41	0.072	1.28E-121	chrX
730	*SLN*	9.71E-126	0.62088596	0.277	0.002	2.85E-121	chr11
727	*SLC52A2*	9.90E-126	0.30779699	0.555	0.168	2.90E-121	chr8
641	*RCN3*	1.87E-125	0.28103353	0.459	0.095	5.48E-121	chr19
580	*POLE4*	3.44E-125	0.33511642	0.598	0.211	1.01E-120	chr2
661	*RPN1*	3.62E-125	0.26757494	0.521	0.141	1.06E-120	chr3
862	*WDR45B*	4.63E-125	0.33391254	0.617	0.211	1.36E-120	chr17
459	*MRPS23*	5.72E-125	0.32214524	0.583	0.195	1.68E-120	chr17
331	*GSDMD*	6.93E-125	0.30370499	0.534	0.151	2.03E-120	chr8
400	*LEPROT*	1.24E-124	0.36927782	0.549	0.175	3.65E-120	chr1
542	*PDHA1*	1.25E-124	0.51113853	0.55	0.192	3.65E-120	chrX
407	*LINC00632*	1.26E-124	0.32007344	0.36	0.045	3.68E-120	chrX
496	*NDUFB1*	1.26E-124	0.42705359	0.702	0.302	3.70E-120	chr14
111	*C7orf50*	2.03E-124	0.34330799	0.629	0.217	5.94E-120	chr7

167	*CKLF*	2.19E-124	0.29131987	0.468	0.11	6.42E-120	chr16
619	*PTK7*	2.49E-124	0.30509733	0.497	0.132	7.29E-120	chr6
560	*PHF5A*	2.77E-124	0.32426211	0.516	0.15	8.13E-120	chr22
188	*COX19*	3.61E-124	0.26371475	0.481	0.114	1.06E-119	chr7
351	*HSF1*	5.83E-124	0.31646577	0.625	0.221	1.71E-119	chr8
89	*C14orf119*	7.67E-124	0.26177199	0.52	0.143	2.25E-119	chr14
332	*GSTK1*	8.41E-124	0.26371272	0.478	0.113	2.47E-119	chr7
348	*HMOX1*	1.14E-123	0.3963545	0.338	0.034	3.34E-119	chr22
631	*RAB3IL1*	1.18E-123	0.25208352	0.368	0.052	3.46E-119	chr11
368	*IGFBP6*	1.52E-123	0.36189437	0.369	0.053	4.47E-119	chr12
673	*S100A13*	2.11E-123	0.56927999	0.728	0.416	6.19E-119	chr1
380	*JTB*	3.23E-123	0.38723414	0.691	0.284	9.46E-119	chr1
625	*PWP1*	4.19E-123	0.26616112	0.508	0.132	1.23E-118	chr12
123	*CCDC107*	4.23E-123	0.27103208	0.501	0.127	1.24E-118	chr9
357	*ICAM1*	5.04E-123	0.28936549	0.285	0.008	1.48E-118	chr19
763	*STARD7*	5.81E-123	0.33772801	0.526	0.166	1.71E-118	chr2
434	*MEAF6*	7.41E-123	0.28874984	0.544	0.165	2.17E-118	chr1
752	*SPPL3*	8.26E-123	0.25294195	0.475	0.112	2.42E-118	chr12
620	*PTN*	1.12E-122	1.01757238	0.784	0.441	3.27E-118	chr7
7	*ACTR1B*	1.45E-122	0.29435018	0.482	0.124	4.26E-118	chr2
726	*SLC44A2*	1.57E-122	0.26677761	0.409	0.075	4.62E-118	chr19
812	*TP53INP1*	3.22E-122	0.26427927	0.314	0.022	9.44E-118	chr8
398	*LAYN*	3.27E-122	0.31484328	0.396	0.064	9.61E-118	chr11
628	*QSOX1*	3.75E-122	0.26234979	0.414	0.076	1.10E-117	chr1
672	*RUNX1T1*	5.09E-122	0.43427001	0.57	0.182	1.49E-117	chr8
312	*GALK1*	5.45E-122	0.35906451	0.585	0.21	1.60E-117	chr17
817	*TRIM55*	6.33E-122	0.3840655	0.266	0	1.86E-117	chr8
229	*DECR1*	1.05E-121	0.42983308	0.619	0.248	3.09E-117	chr8
221	*DCAF13*	1.16E-121	0.41688846	0.666	0.286	3.41E-117	chr8
259	*EIF3B*	2.37E-121	0.37335294	0.705	0.302	6.97E-117	chr7
718	*SKP1*	2.54E-121	0.51060017	0.806	0.486	7.45E-117	chr5
860	*WDR34*	3.70E-121	0.25327908	0.38	0.063	1.09E-116	chr9
127	*CCL2*	3.89E-121	0.56891003	0.356	0.047	1.14E-116	chr17
37	*AQP1*	4.03E-121	0.65008988	0.366	0.052	1.18E-116	chr7
144	*CDCA4*	7.63E-121	0.29683765	0.464	0.113	2.24E-116	chr14
506	*NKAIN4*	7.76E-121	0.32923857	0.348	0.043	2.28E-116	chr20
809	*TNNI1*	9.06E-121	0.55932782	0.281	0.009	2.66E-116	chr1
72	*B3GALT6*	1.54E-120	0.26451719	0.438	0.097	4.52E-116	chr1
297	*FCGRT*	1.98E-120	0.37893309	0.613	0.218	5.80E-116	chr19
21	*ALKBH7*	2.29E-120	0.28699026	0.584	0.191	6.71E-116	chr19

202	*CRNDE*	2.65E-120	0.32167075	0.532	0.159	7.78E-116	chr16
820	*TROVE2*	3.22E-120	0.29177709	0.518	0.149	9.45E-116	chr1
636	*RARRES2*	3.64E-120	0.53174037	0.64	0.253	1.07E-115	chr7
248	*EBPL*	6.94E-120	0.31320648	0.524	0.153	2.04E-115	chr13
73	*B4GALT2*	8.27E-120	0.27588745	0.487	0.126	2.43E-115	chr1
300	*FHL1*	9.57E-120	0.26561592	0.448	0.1	2.81E-115	chrX
487	*NCALD*	1.13E-119	0.3656608	0.333	0.039	3.33E-115	chr8
591	*PPDPF*	1.30E-119	0.47434451	0.746	0.347	3.81E-115	chr20
691	*SEC61A1*	1.37E-119	0.35216794	0.598	0.21	4.02E-115	chr3
335	*HADHB*	1.63E-119	0.3255743	0.591	0.209	4.77E-115	chr2
43	*ARMC1*	1.63E-119	0.35407454	0.546	0.172	4.78E-115	chr8
63	*ATP6AP2*	1.64E-119	0.28676974	0.608	0.218	4.81E-115	chrX
378	*JAGN1*	1.71E-119	0.3573451	0.525	0.166	5.00E-115	chr3
714	*SIGIRR*	3.70E-119	0.32772507	0.487	0.136	1.08E-114	chr11
11	*AEBP1*	4.03E-119	0.62091479	0.705	0.299	1.18E-114	chr7
568	*PLAC9*	1.11E-118	0.62287426	0.407	0.094	3.27E-114	chr10
799	*TMEM45A*	1.32E-118	0.29736002	0.399	0.075	3.88E-114	chr3
35	*APMAP*	1.33E-118	0.26023938	0.472	0.12	3.91E-114	chr20
651	*RNF181*	1.70E-118	0.32258845	0.669	0.265	4.98E-114	chr2
256	*EGLN3*	1.70E-118	0.37208636	0.403	0.075	4.99E-114	chr14
688	*SDHC*	3.29E-118	0.32698587	0.622	0.228	9.64E-114	chr1
866	*XBP1*	4.76E-118	0.39089945	0.637	0.242	1.40E-113	chr22
590	*PPCS*	7.21E-118	0.26603082	0.498	0.135	2.12E-113	chr1
87	*C12orf75*	7.73E-118	0.28946587	0.441	0.101	2.27E-113	chr12
533	*OXR1*	9.08E-118	0.32488472	0.493	0.132	2.66E-113	chr8
495	*NDUFA5*	9.09E-118	0.27874628	0.637	0.223	2.67E-113	chr7
396	*LAMTOR5*	1.10E-117	0.46845856	0.711	0.365	3.23E-113	chr1
227	*DDX21*	1.24E-117	0.37286533	0.618	0.231	3.63E-113	chr10
5	*ACTC1*	1.53E-117	0.73105508	0.295	0.019	4.48E-113	chr15
160	*CHRND*	2.28E-117	0.40695594	0.258	0	6.70E-113	chr2
458	*MRPS18B*	2.90E-117	0.27605427	0.622	0.224	8.52E-113	chr6
729	*SLIRP*	3.55E-117	0.40200214	0.722	0.343	1.04E-112	chr14
19	*ALDH1A3*	4.71E-117	0.37172784	0.433	0.098	1.38E-112	chr15
543	*PDIA4*	5.12E-117	0.3176121	0.623	0.228	1.50E-112	chr7
310	*GADD45A*	6.11E-117	0.51346934	0.583	0.22	1.79E-112	chr1
319	*GLO1*	6.31E-117	0.31643719	0.633	0.237	1.85E-112	chr6
317	*GIP*	6.66E-117	0.49029567	0.258	0.001	1.95E-112	chr17
307	*FUOM*	1.03E-116	0.2667405	0.443	0.103	3.02E-112	chr10
550	*PEPD*	1.32E-116	0.28091405	0.5	0.143	3.87E-112	chr19
93	*C17orf58*	1.91E-116	0.29461311	0.389	0.074	5.60E-112	chr17

333	*GTF3A*	3.35E-116	0.36718431	0.644	0.258	9.83E-112	chr13
128	*CCM2*	6.59E-116	0.286897	0.56	0.182	1.93E-111	chr7
45	*ASNA1*	1.18E-115	0.25253487	0.515	0.155	3.48E-111	chr19
341	*HK1*	2.13E-115	0.25788065	0.463	0.114	6.25E-111	chr10
146	*CDK16*	3.10E-115	0.26613533	0.473	0.128	9.10E-111	chrX
232	*DGAT1*	3.36E-115	0.32708096	0.525	0.155	9.86E-111	chr8
650	*RNASEH2C*	6.82E-115	0.28608306	0.529	0.165	2.00E-110	chr11
825	*TTYH3*	1.39E-114	0.29996026	0.428	0.094	4.06E-110	chr7
720	*SLC25A37*	1.97E-114	0.45091497	0.477	0.127	5.79E-110	chr8
800	*TMEM50A*	7.99E-114	0.33642867	0.64	0.241	2.35E-109	chr1
592	*PPIC*	8.16E-114	0.29690556	0.525	0.164	2.39E-109	chr5
32	*AP3D1*	8.85E-114	0.27026358	0.554	0.179	2.60E-109	chr19
404	*LIMA1*	9.47E-114	0.28392685	0.482	0.13	2.78E-109	chr12
611	*PSMB8*	1.72E-113	0.37263448	0.571	0.203	5.03E-109	chr6
824	*TSPO*	2.51E-113	0.48153527	0.747	0.405	7.35E-109	chr22
666	*RRAGC*	6.31E-113	0.39494378	0.495	0.155	1.85E-108	chr1
723	*SLC39A1*	9.02E-113	0.31180729	0.608	0.231	2.65E-108	chr1
34	*APH1A*	9.16E-113	0.33017187	0.65	0.253	2.69E-108	chr1
142	*CDC25B*	9.42E-113	0.26243613	0.375	0.064	2.76E-108	chr20
390	*KRT17*	1.02E-112	1.02187924	0.318	0.038	2.98E-108	chr17
515	*NPY*	4.52E-112	0.48513533	0.425	0.103	1.33E-107	chr7
804	*TMEM98*	4.57E-112	0.34271902	0.625	0.245	1.34E-107	chr17
77	*BLCAP*	5.48E-112	0.2584717	0.492	0.137	1.61E-107	chr20
521	*NUCB2*	1.61E-111	0.34465801	0.551	0.188	4.72E-107	chr11
402	*LGALS3BP*	2.05E-111	0.35971541	0.323	0.039	6.00E-107	chr17
519	*NTRK2*	2.21E-111	0.34924625	0.295	0.022	6.49E-107	chr9
243	*DUSP1*	2.58E-111	0.63553839	0.663	0.339	7.58E-107	chr5
507	*NME3*	3.59E-111	0.28578891	0.351	0.054	1.05E-106	chr16
446	*MMADHC*	4.40E-111	0.25509047	0.592	0.206	1.29E-106	chr2
593	*PPP1CB*	7.01E-111	0.32360113	0.62	0.239	2.06E-106	chr2
608	*PSMA1*	7.71E-111	0.31367778	0.707	0.328	2.26E-106	chr11
847	*UXT*	9.45E-111	0.3235679	0.656	0.27	2.77E-106	chrX
124	*CCDC167*	1.04E-110	0.31704599	0.583	0.213	3.06E-106	chr6
86	*C11orf96*	1.66E-110	0.62313436	0.407	0.102	4.87E-106	chr11
38	*ARF3*	1.89E-110	0.25124079	0.436	0.107	5.56E-106	chr12
474	*MYADM*	2.17E-110	0.41454579	0.475	0.132	6.37E-106	chr19
703	*SERPINI1*	4.64E-110	0.34599199	0.355	0.061	1.36E-105	chr3
412	*LOX*	6.66E-110	0.27857919	0.345	0.05	1.95E-105	chr5
453	*MRPL33*	8.71E-110	0.3030409	0.637	0.238	2.55E-105	chr2
376	*ITGAE*	9.66E-110	0.26296731	0.533	0.167	2.83E-105	chr17

881	*ZNF706*	1.71E-109	0.47456604	0.762	0.453	5.02E-105	chr8
387	*KLC1*	1.74E-109	0.26338955	0.493	0.138	5.10E-105	chr14
880	*ZNF593*	2.12E-109	0.32971029	0.624	0.259	6.21E-105	chr1
513	*NPTX2*	2.47E-109	0.49435309	0.551	0.193	7.24E-105	chr7
485	*NAGLU*	2.68E-109	0.3035461	0.41	0.094	7.87E-105	chr17
526	*OAT*	4.18E-109	0.25419253	0.458	0.121	1.23E-104	chr10
520	*NUCB1*	7.61E-109	0.25040093	0.51	0.151	2.23E-104	chr19
143	*CDC37*	1.17E-108	0.33651508	0.691	0.308	3.43E-104	chr19
260	*EIF3L*	1.64E-108	0.4311113	0.765	0.451	4.82E-104	chr22
877	*ZNF22*	1.97E-108	0.26934973	0.575	0.205	5.78E-104	chr10
746	*SPATS2L*	2.44E-108	0.33606548	0.705	0.31	7.16E-104	chr2
462	*MRPS7*	3.20E-108	0.38531002	0.636	0.285	9.38E-104	chr17
318	*GIPC1*	3.47E-108	0.28447141	0.534	0.187	1.02E-103	chr19
874	*ZFAND2A*	6.73E-108	0.39705897	0.597	0.229	1.97E-103	chr7
360	*IER2*	8.48E-108	0.5242587	0.767	0.391	2.49E-103	chr19
293	*FBXO17*	9.34E-108	0.25632958	0.491	0.143	2.74E-103	chr19
731	*SMARCB1*	1.05E-107	0.35137064	0.613	0.251	3.08E-103	chr22
324	*GNPDA1*	1.42E-107	0.31471061	0.451	0.125	4.16E-103	chr5
129	*CCNB1IP1*	1.62E-107	0.26821442	0.62	0.229	4.76E-103	chr14
629	*RAB31*	1.82E-107	0.30033319	0.424	0.104	5.34E-103	chr18
122	*CBX6*	2.37E-107	0.26771339	0.518	0.161	6.96E-103	chr22
573	*PLIN3*	2.56E-107	0.25752489	0.525	0.162	7.50E-103	chr19
216	*CYC1*	4.72E-107	0.4732586	0.771	0.515	1.39E-102	chr8
379	*JPX*	4.86E-107	0.28616381	0.409	0.092	1.43E-102	chrX
383	*KDELR1*	4.99E-107	0.39439405	0.755	0.382	1.46E-102	chr19
698	*9-Sep*	8.98E-107	0.34105592	0.577	0.218	2.63E-102	chr17
240	*DPP7*	9.31E-107	0.29371644	0.567	0.194	2.73E-102	chr9
468	*MT1E*	9.40E-107	0.42132569	0.571	0.19	2.76E-102	chr16
171	*CLU*	1.61E-106	0.36161339	0.521	0.167	4.72E-102	chr8
238	*DNAJB11*	2.34E-106	0.28978622	0.568	0.197	6.85E-102	chr3
286	*FAM213A*	2.84E-106	1.33202173	0.493	0.205	8.34E-102	chr10
195	*CPNE1*	2.96E-106	0.25260345	0.623	0.232	8.69E-102	chr20
6	*ACTN4*	3.32E-106	0.29145105	0.6	0.232	9.74E-102	chr19
366	*IGFBP4*	4.09E-106	0.44145894	0.57	0.213	1.20E-101	chr17
571	*PLEC*	5.25E-106	0.37172179	0.543	0.174	1.54E-101	chr8
644	*RGS4*	1.11E-105	0.5198206	0.301	0.031	3.25E-101	chr1
584	*POLR2L*	2.61E-105	0.50282792	0.785	0.494	7.66E-101	chr11
289	*FAM89B*	3.85E-105	0.2920956	0.585	0.216	1.13E-100	chr11
676	*SAE1*	5.14E-105	0.27578447	0.535	0.183	1.51E-100	chr19
246	*DUT*	1.32E-104	0.25661561	0.597	0.212	3.88E-100	chr15

577	*PLTP*	1.88E-104	0.26732894	0.393	0.089	5.52E-100	chr20
490	*NDUFA1*	2.86E-104	0.33657215	0.685	0.284	8.40E-100	chrX
423	*LYPLA2*	4.89E-104	0.25071707	0.6	0.229	1.43E-99	chr1
120	*CAST*	6.04E-104	0.27038126	0.521	0.163	1.77E-99	chr5
546	*PDPN*	7.17E-104	0.31137133	0.352	0.067	2.10E-99	chr1
80	*BOP1*	1.70E-103	0.32177893	0.636	0.259	4.99E-99	chr8
359	*IDS*	3.03E-103	0.25784295	0.351	0.061	8.88E-99	chrX
211	*CXCL3*	3.27E-103	0.35206604	0.261	0.016	9.59E-99	chr4
68	*ATXN10*	3.79E-103	0.30621511	0.583	0.224	1.11E-98	chr22
707	*SH3BP5*	4.79E-103	0.33145325	0.465	0.144	1.40E-98	chr3
589	*PPA1*	9.79E-103	0.25970849	0.6	0.232	2.87E-98	chr10
96	*C18orf32*	1.06E-102	0.2690512	0.367	0.072	3.10E-98	chr18
218	*CYTL1*	1.10E-102	0.57827051	0.589	0.236	3.23E-98	chr4
579	*PMAIP1*	1.36E-102	0.35748208	0.556	0.192	3.98E-98	chr18
632	*RAB42*	1.44E-102	0.38712209	0.35	0.065	4.23E-98	chr1
435	*MED10*	2.53E-102	0.28514129	0.627	0.247	7.43E-98	chr5
69	*AURKAIP1*	2.89E-102	0.31918737	0.719	0.344	8.49E-98	chr1
395	*LAMTOR1*	3.09E-102	0.45202051	0.688	0.368	9.07E-98	chr11
39	*ARHGDIA*	3.19E-102	0.33946724	0.683	0.328	9.37E-98	chr17
234	*DGUOK*	3.78E-102	0.31250768	0.708	0.313	1.11E-97	chr2
44	*ASAH1*	4.57E-102	0.32620274	0.445	0.127	1.34E-97	chr8
254	*EGFR*	5.01E-102	0.46300721	0.564	0.201	1.47E-97	chr7
565	*PITX3*	5.17E-102	0.33706096	0.254	0.014	1.52E-97	chr10
725	*SLC40A1*	1.70E-101	0.4091157	0.415	0.117	4.99E-97	chr2
340	*HIST1H2BG*	1.71E-101	0.25352755	0.349	0.06	5.01E-97	chr6
547	*PDXK*	2.00E-101	0.26918781	0.545	0.188	5.86E-97	chr21
125	*CCDC34*	8.61E-101	0.30935677	0.502	0.167	2.53E-96	chr11
164	*CITED1*	9.75E-101	0.26760119	0.275	0.025	2.86E-96	chrX
518	*NSMCE2*	1.06E-100	0.25798201	0.542	0.184	3.10E-96	chr8
854	*VKORC1*	1.06E-100	0.31612762	0.632	0.261	3.12E-96	chr16
193	*COX7B*	2.38E-100	0.39049728	0.72	0.359	6.99E-96	chrX
249	*ECH1*	5.09E-100	0.29158586	0.635	0.269	1.49E-95	chr19
177	*COL13A1*	7.92E-100	0.26818545	0.359	0.069	2.32E-95	chr10
769	*SURF4*	1.12E-99	0.32850953	0.648	0.28	3.27E-95	chr9
451	*MRC2*	1.29E-99	0.36638201	0.561	0.203	3.79E-95	chr17
311	*GADD45GIP1*	2.27E-99	0.32390335	0.755	0.378	6.66E-95	chr19
844	*USP11*	2.85E-99	0.25001435	0.511	0.169	8.36E-95	chrX
516	*NR4A1*	1.46E-98	0.2838621	0.569	0.222	4.27E-94	chr12
172	*CMTM3*	2.57E-98	0.25014255	0.525	0.175	7.54E-94	chr16
414	*LOXL2*	2.58E-98	0.4135302	0.496	0.163	7.58E-94	chr8

823	*TSPAN5*	7.81E-98	0.3065709	0.507	0.171	2.29E-93	chr4
623	*PTRHD1*	8.42E-98	0.30026437	0.633	0.278	2.47E-93	chr2
749	*SPINK6*	1.18E-97	1.35318327	0.313	0.056	3.45E-93	chr5
819	*TRMT112*	1.30E-97	0.35774882	0.788	0.432	3.81E-93	chr11
583	*POLR2K*	1.43E-97	0.38814873	0.755	0.435	4.19E-93	chr8
306	*FSCN1*	3.55E-97	0.40558521	0.775	0.399	1.04E-92	chr7
871	*ZC2HC1A*	5.18E-97	0.26132296	0.443	0.128	1.52E-92	chr8
252	*EFR3A*	1.01E-96	0.25184513	0.413	0.105	2.96E-92	chr8
337	*HEY1*	1.17E-96	0.48613003	0.424	0.13	3.44E-92	chr8
551	*PERP*	7.74E-96	0.36487868	0.526	0.191	2.27E-91	chr6
81	*BRI3*	1.24E-95	0.43358093	0.755	0.446	3.64E-91	chr7
833	*TXNL4A*	1.48E-95	0.250918	0.674	0.301	4.34E-91	chr18
832	*TXNIP*	1.53E-95	0.33175435	0.434	0.119	4.50E-91	chr1
198	*CPVL*	2.30E-95	0.39451179	0.396	0.111	6.75E-91	chr7
612	*PSMD13*	2.47E-95	0.26352585	0.623	0.259	7.26E-91	chr11
411	*LMNA*	4.09E-95	0.51368847	0.811	0.521	1.20E-90	chr1
278	*EVA1B*	4.39E-95	0.31062862	0.648	0.3	1.29E-90	chr1
17	*AKR1B1*	5.48E-95	0.43277198	0.565	0.241	1.61E-90	chr7
475	*MYC*	7.49E-95	0.26418671	0.485	0.151	2.20E-90	chr8
782	*TMCO1*	8.00E-95	0.25432352	0.666	0.27	2.35E-90	chr1
401	*LGALS1*	1.38E-94	0.32048228	0.888	0.639	4.04E-90	chr22
724	*SLC3A2*	2.25E-94	0.39827842	0.671	0.31	6.61E-90	chr11
262	*EIF4EBP1*	2.29E-94	0.33339591	0.648	0.305	6.71E-90	chr8
554	*PGD*	2.82E-94	0.26333057	0.51	0.181	8.28E-90	chr1
373	*INSIG1*	2.92E-94	0.32135479	0.43	0.124	8.57E-90	chr7
117	*CALU*	3.27E-94	0.41108136	0.739	0.382	9.61E-90	chr7
669	*RTCB*	3.96E-94	0.25328045	0.552	0.215	1.16E-89	chr22
478	*MYL6B*	4.88E-94	0.31091486	0.704	0.318	1.43E-89	chr12
665	*RPUSD3*	6.83E-94	0.30210737	0.442	0.137	2.00E-89	chr3
635	*RAPSN*	8.80E-94	0.34807663	0.267	0.026	2.58E-89	chr11
837	*UQCR10*	3.53E-93	0.52974874	0.746	0.461	1.03E-88	chr22
375	*ITGA10*	6.61E-93	0.40617181	0.278	0.034	1.94E-88	chr1
358	*ID1*	6.76E-93	0.51319113	0.675	0.332	1.98E-88	chr20
835	*UBE2T*	7.53E-93	0.28077429	0.399	0.109	2.21E-88	chr1
347	*HMGN2*	2.18E-92	0.60068381	0.822	0.574	6.39E-88	chr1
711	*SHISA2*	3.52E-92	0.43750749	0.388	0.113	1.03E-87	chr13
639	*RBM38*	5.20E-92	0.38545566	0.372	0.101	1.53E-87	chr20
850	*VAT1*	5.96E-92	0.29030881	0.42	0.126	1.75E-87	chr17
483	*N4BP2L2*	6.37E-92	0.36062331	0.654	0.263	1.87E-87	chr13
849	*VASN*	8.45E-92	0.26494612	0.381	0.095	2.48E-87	chr16

511	*NPDC1*	9.26E-92	0.26472142	0.345	0.074	2.72E-87	chr9
316	*GHITM*	6.16E-91	0.30765638	0.622	0.291	1.81E-86	chr10
758	*SSR1*	1.03E-90	0.2616198	0.599	0.241	3.02E-86	chr6
609	*PSMA2*	6.05E-90	0.29671456	0.764	0.425	1.77E-85	chr7
537	*PABPC4*	6.85E-90	0.27532004	0.652	0.279	2.01E-85	chr1
827	*TUFM*	2.13E-89	0.26018688	0.697	0.312	6.24E-85	chr16
572	*PLEKHA1*	3.36E-89	0.26068733	0.42	0.116	9.86E-85	chr10
199	*CRABP2*	4.06E-89	0.44007515	0.713	0.349	1.19E-84	chr1
722	*SLC2A3*	4.22E-89	0.4774193	0.404	0.116	1.24E-84	chr12
701	*SERPINF1*	5.44E-89	0.33664432	0.641	0.305	1.60E-84	chr17
786	*TMED4*	1.24E-88	0.25977241	0.588	0.225	3.65E-84	chr7
530	*OSR2*	4.39E-88	0.30473969	0.419	0.124	1.29E-83	chr8
613	*PSMD3*	7.14E-88	0.27834353	0.622	0.272	2.10E-83	chr17
646	*RHOB*	8.21E-88	0.37378569	0.657	0.326	2.41E-83	chr2
791	*TMEM14B*	1.13E-87	0.26454752	0.616	0.245	3.32E-83	chr6
811	*TP53I3*	1.58E-87	0.28176196	0.465	0.165	4.65E-83	chr2
344	*HLA-C*	2.24E-87	0.47901617	0.72	0.355	6.58E-83	chr6
209	*CTSH*	2.63E-87	0.2624694	0.363	0.091	7.70E-83	chr15
141	*CDC20*	7.55E-87	0.346683	0.447	0.143	2.22E-82	chr1
728	*SLC5A3*	7.58E-87	0.86367505	0.518	0.194	2.22E-82	chr21
205	*CTGF*	1.39E-86	0.50093255	0.586	0.277	4.09E-82	chr6
161	*CIRBP*	1.60E-86	0.38873687	0.868	0.516	4.68E-82	chr19
445	*MLLT11*	2.95E-86	0.28877296	0.515	0.195	8.65E-82	chr1
138	*CD81*	3.85E-86	0.58838521	0.706	0.378	1.13E-81	chr11
489	*NDRG1*	4.07E-86	0.91826653	0.52	0.199	1.20E-81	chr8
277	*ERP29*	4.21E-86	0.30065163	0.699	0.374	1.24E-81	chr12
33	*APEX1*	6.21E-86	0.32036624	0.705	0.358	1.82E-81	chr14
267	*EMC3*	1.43E-85	0.25337436	0.506	0.184	4.18E-81	chr3
220	*DBI*	5.20E-85	0.36175936	0.749	0.39	1.53E-80	chr2
67	*ATRAID*	7.47E-85	0.33160652	0.728	0.391	2.19E-80	chr2
634	*RALY*	1.06E-84	0.30691028	0.669	0.329	3.11E-80	chr20
431	*MDFI*	2.72E-84	0.28464869	0.668	0.309	7.99E-80	chr6
322	*GLUL*	4.19E-84	0.40170659	0.611	0.305	1.23E-79	chr1
447	*MMP2*	1.86E-83	0.32285649	0.591	0.255	5.47E-79	chr16
739	*SNHG9*	2.33E-83	0.2935173	0.339	0.08	6.82E-79	chr16
22	*ANAPC11*	2.81E-83	0.40087632	0.764	0.498	8.24E-79	chr17
168	*CKS1B*	4.62E-82	0.30208198	0.667	0.32	1.35E-77	chr1
391	*KRT8*	8.73E-82	0.34781566	0.274	0.044	2.56E-77	chr12
433	*MDM4*	1.31E-81	0.27141155	0.393	0.11	3.83E-77	chr1
558	*PGM2L1*	1.56E-81	0.27793459	0.446	0.149	4.58E-77	chr11

223	*DDIT3*	4.56E-81	0.36742263	0.594	0.277	1.34E-76	chr12
498	*NDUFB4*	1.24E-80	0.25291088	0.703	0.333	3.63E-76	chr3
196	*CPNE3*	1.31E-80	0.2652684	0.62	0.287	3.83E-76	chr8
314	*GDF15*	1.38E-80	0.81638424	0.254	0.033	4.04E-76	chr19
538	*PARK7*	1.64E-80	0.30614505	0.796	0.511	4.82E-76	chr1
834	*UBE2C*	1.67E-80	0.34078205	0.464	0.162	4.90E-76	chr20
145	*CDK1*	2.75E-80	0.28850331	0.351	0.091	8.06E-76	chr10
253	*EGFL7*	2.78E-80	0.26502812	0.517	0.193	8.15E-76	chr9
765	*STOML2*	3.34E-80	0.25481042	0.662	0.329	9.81E-76	chr9
744	*SPARCL1*	4.28E-80	0.80379769	0.347	0.091	1.26E-75	chr4
602	*PRKDC*	6.85E-80	0.2526254	0.625	0.268	2.01E-75	chr8
236	*DLC1*	7.96E-80	0.28295638	0.391	0.115	2.33E-75	chr8
178	*COL4A2*	2.61E-79	0.47067666	0.612	0.267	7.66E-75	chr13
500	*NDUFB8*	3.04E-78	0.3216737	0.731	0.433	8.91E-74	chr10
753	*SQLE*	5.51E-78	0.33557043	0.611	0.267	1.62E-73	chr8
320	*GLRX5*	1.53E-77	0.25231773	0.63	0.298	4.48E-73	chr14
783	*TMED10*	1.37E-76	0.25911295	0.727	0.402	4.01E-72	chr14
269	*EMP2*	4.74E-76	0.26776297	0.541	0.213	1.39E-71	chr16
191	*COX7A2*	1.09E-75	0.31068257	0.806	0.478	3.19E-71	chr6
382	*KCNMA1*	1.38E-75	0.29463406	0.26	0.041	4.06E-71	chr10
76	*BIRC5*	3.03E-75	0.32979872	0.458	0.17	8.90E-71	chr17
690	*SEC13*	3.95E-75	0.34671875	0.609	0.316	1.16E-70	chr3
26	*ANKRD37*	1.00E-74	0.29808221	0.349	0.093	2.94E-70	chr4
308	*FXYD5*	3.11E-74	0.37836351	0.452	0.191	9.11E-70	chr19
428	*MAP1LC3B*	3.52E-74	0.25468348	0.644	0.289	1.03E-69	chr16
31	*AP2M1*	4.61E-74	0.35064139	0.709	0.44	1.35E-69	chr3
273	*ENY2*	6.71E-74	0.42581353	0.831	0.627	1.97E-69	chr8
606	*PRSS35*	9.66E-74	0.28965109	0.305	0.072	2.83E-69	chr6
642	*RGS16*	1.92E-73	0.29160311	0.483	0.187	5.63E-69	chr1
581	*POLR2G*	4.71E-73	0.25885661	0.649	0.341	1.38E-68	chr11
425	*MAGED1*	1.13E-72	0.28347046	0.683	0.372	3.32E-68	chrX
870	*YWHAZ*	1.15E-72	0.38499981	0.832	0.619	3.38E-68	chr8
14	*AHI1*	1.44E-72	0.25459424	0.457	0.164	4.22E-68	chr6
878	*ZNF395*	6.53E-71	0.34410065	0.291	0.067	1.92E-66	chr8
808	*TNMD*	7.16E-70	0.79846137	0.318	0.09	2.10E-65	chrX
539	*PARP1*	1.43E-69	0.41491732	0.571	0.29	4.19E-65	chr1
760	*SSR3*	1.69E-69	0.26943238	0.671	0.329	4.95E-65	chr3
559	*PHF14*	1.69E-69	0.2512346	0.718	0.363	4.96E-65	chr7
681	*SCD*	4.90E-69	0.27231687	0.408	0.143	1.44E-64	chr10
346	*HM13*	5.92E-69	0.25685368	0.663	0.328	1.74E-64	chr20

242	*DSTN*	2.77E-68	0.27277176	0.724	0.372	8.11E-64	chr20
261	*EIF4A3*	9.79E-68	0.27236874	0.605	0.304	2.87E-63	chr17
575	*PLOD2*	2.47E-67	0.267225	0.479	0.189	7.24E-63	chr3
27	*ANXA1*	1.42E-66	0.41234665	0.556	0.255	4.15E-62	chr9
671	*RTN4*	2.12E-66	0.37519553	0.77	0.48	6.22E-62	chr2
294	*FBXO32*	3.34E-66	0.25576532	0.266	0.057	9.79E-62	chr8
541	*PDGFA*	2.00E-65	0.27410205	0.265	0.057	5.86E-61	chr7
292	*FBN2*	8.51E-64	0.3519841	0.419	0.163	2.50E-59	chr5
610	*PSMB7*	5.08E-63	0.25054858	0.714	0.478	1.49E-58	chr9
116	*CALD1*	7.59E-63	0.39232277	0.843	0.543	2.23E-58	chr7
352	*HSPA1A*	8.48E-63	0.33750138	0.64	0.395	2.49E-58	chr6
633	*RAD21*	1.19E-62	0.29240767	0.762	0.448	3.49E-58	chr8
637	*RBFOX2*	1.32E-62	0.29482511	0.672	0.366	3.87E-58	chr22
417	*LTBP1*	1.57E-62	0.33271932	0.499	0.216	4.61E-58	chr2
742	*SOCS3*	2.38E-62	0.28670748	0.409	0.153	6.98E-58	chr17
245	*DUSP6*	1.38E-61	0.36856359	0.597	0.321	4.06E-57	chr12
527	*OGN*	1.98E-60	0.51425918	0.335	0.113	5.81E-56	chr9
776	*TCF25*	2.90E-60	0.2570368	0.722	0.394	8.50E-56	chr16
104	*C1R*	3.99E-60	0.25644458	0.348	0.12	1.17E-55	chr12
175	*COCH*	7.86E-60	0.26505675	0.32	0.103	2.31E-55	chr14
761	*ST13*	2.62E-59	0.2801003	0.771	0.5	7.69E-55	chr22
813	*TPI1*	3.25E-59	0.3434244	0.873	0.626	9.52E-55	chr12
585	*POMP*	3.40E-59	0.28156171	0.796	0.587	9.99E-55	chr13
869	*YBX3*	3.25E-58	0.30737028	0.769	0.492	9.54E-54	chr12
276	*ERH*	3.98E-58	0.26170829	0.774	0.525	1.17E-53	chr14
268	*EMILIN1*	5.37E-58	0.25636477	0.625	0.307	1.58E-53	chr2
166	*CKB*	6.27E-58	0.46625907	0.674	0.407	1.84E-53	chr14
864	*WIF1*	9.70E-58	0.28724417	0.339	0.118	2.85E-53	chr12
272	*ENPP2*	3.88E-55	0.40501273	0.447	0.201	1.14E-50	chr8
467	*MT-ND6*	5.89E-54	0.27084886	0.613	0.298	1.73E-49	chrM
600	*PRDX6*	6.63E-54	0.36805847	0.725	0.471	1.94E-49	chr1
389	*KPNA2*	1.21E-53	0.41428464	0.575	0.312	3.55E-49	chr17
271	*ENO2*	1.33E-52	0.26306416	0.323	0.114	3.90E-48	chr12
734	*SMS*	4.87E-52	0.25150981	0.521	0.268	1.43E-47	chrX
364	*IFITM3*	1.83E-50	0.28580437	0.784	0.54	5.38E-46	chr11
674	*S100A16*	8.75E-50	0.36530663	0.38	0.171	2.57E-45	chr1
664	*RPS26*	1.88E-49	0.28822766	0.868	0.656	5.52E-45	chr12
176	*COL11A1*	5.73E-49	0.31141224	0.521	0.262	1.68E-44	chr1
517	*NRN1*	1.26E-39	0.3225009	0.409	0.199	3.70E-35	chr6
464	*MT-ND2*	1.62E-37	0.56552153	0.933	0.727	4.76E-33	chrM

**865**	***WSB1***	**1.05E-36**	**0.29302085**	**0.739**	**0.415**	**3.09E-32**	**chr17**
**852**	***VEGFA***	**1.18E-36**	**0.77711692**	**0.456**	**0.249**	**3.47E-32**	**chr6**
**514**	***NPW***	**1.11E-34**	**0.25087623**	**0.726**	**0.422**	**3.25E-30**	**chr16**
**821**	***TRPS1***	**2.33E-24**	**0.25820324**	**0.613**	**0.359**	**6.85E-20**	**chr8**
**466**	***MT-ND5***	**2.28E-21**	**0.44677319**	**0.903**	**0.649**	**6.68E-17**	**chrM**
**367**	***IGFBP5***	**5.12E-17**	**0.27529091**	**0.724**	**0.453**	**1.50E-12**	**chr2**
**465**	***MT-ND4L***	**1.07E-10**	**0.33910135**	**0.755**	**0.499**	**3.14E-06**	**chrM**

Supplemental Table 4: Marker genes of cells that demonstrate gain of Chr8q.
